# Ionic Liquid–Liquid Chromatography: A New General Purpose Separation Methodology

**DOI:** 10.1007/s41061-017-0159-y

**Published:** 2017-08-10

**Authors:** Leslie Brown, Martyn J. Earle, Manuela A. Gîlea, Natalia V. Plechkova, Kenneth R. Seddon

**Affiliations:** 1 AECS-QuikPrep Ltd, 55 Gower Street, London, WC1 6HQ UK; 2 The QUILL Research Centre, School of Chemistry, The Queen’s University of Belfast, Stranmillis Road, Belfast, Northern Ireland BT9 5AG UK

**Keywords:** Ionic liquids, Countercurrent chromatography, Ionic liquid–liquid chromatography, Separations, Solvent engineering

## Abstract

Ionic liquids can form biphasic solvent systems with many organic solvents and water, and these solvent systems can be used in liquid–liquid separations and countercurrent chromatography. The wide range of ionic liquids that can by synthesised, with specifically tailored properties, represents a new philosophy for the separation of organic, inorganic and bio-based materials. A customised countercurrent chromatograph has been designed and constructed specifically to allow the more viscous character of ionic liquid-based solvent systems to be used in a wide variety of separations (including transition metal salts, arenes, alkenes, alkanes, bio-oils and sugars).

## Introduction

Over the last two decades, there have been considerable advances in the science of liquid–liquid chromatography (LLC) [1, 2] and its variant, countercurrent chromatography (CCC), with the latter considered to be a continuous, automated form of liquid–liquid separation [3]. These techniques are based on the ability of solutes to distribute themselves between two mutually immiscible phases: a mobile liquid phase (MP) and a stationary liquid phase (SP) [4]. LLC sits at the interface of three areas of research: chemical engineering [5], solvent engineering [6, 7] and chromatographic science [1]. In this chapter, we use the term solvent engineering in the sense of design, production and use of a solvent (or solvent system) to give enhanced control, performance, yield and/or selectivity in physical, chemical or biochemical reactions, processes and separations. The evolution of CCC instrumentation, in large part carried out by Ito [8], has allowed higher stationary phase retention, which in turn generates better resolution, faster separations and continuous processes with industrial applicability [9].

The use of large amounts of mixed organic solvents in LLC is usually associated with difficulties in the recycling of solvents [10] and the release of volatile organic compounds (VOCs) [11]. The option of solvent recycling [12] allows the reuse of the column effluent, but the full separation of effluents into single solvent components is a cumbersome and expensive process due to the similarity in boiling points of individual components (with potential azeotrope formation in some cases) [13, 14]. However, in spite of these limitations, industrial processes based on LLC have been described [9, 15, 16]. In order to expand the range of stationary and mobile phases available in CCC and related technologies and to make use of the unique and greater dissolution capabilities of ionic liquids [17], ionic liquids have been examined as new and alternative solvents in CCC.

**Fig. 1:**
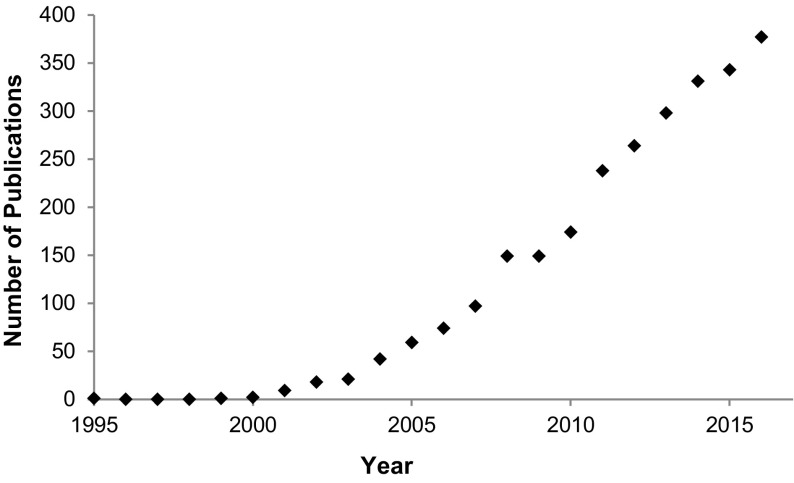
The number of journal articles based on a Web of Science [18] search of the terms “ionic liquid” and “chromatography” for the time period 1995–2016

Ionic liquids have been described as “designer solvents”, as they can be engineered to solve specific problems, such as the dissolution of a large range of materials, replace toxic and volatile solvents, produce multiphasic solvent systems to enhance separation efficiency or allow simplified separations [19]. Their use in chromatographic separations has greatly increased since their first use in 1999 [20], and the growth in the number of publications is shown in Fig. 1.

In this chapter, we demonstrate that a combination of solvent engineering and chromatographic and analytical techniques can be used to enable a diverse range of separations, including inorganic, organic, biochemical and petrochemical applications [21].

## Introduction to Countercurrent Chromatography

**Fig. 2:**
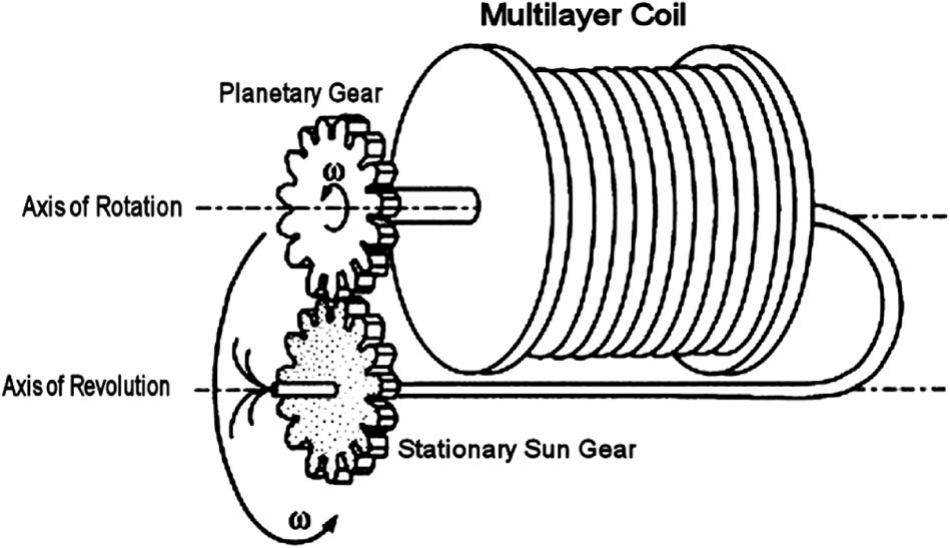
Schematic of the J-type synchronous planetary motion centrifuge used in countercurrent chromatography [8]. Granted with the permission from Wiley

**Fig. 3:**
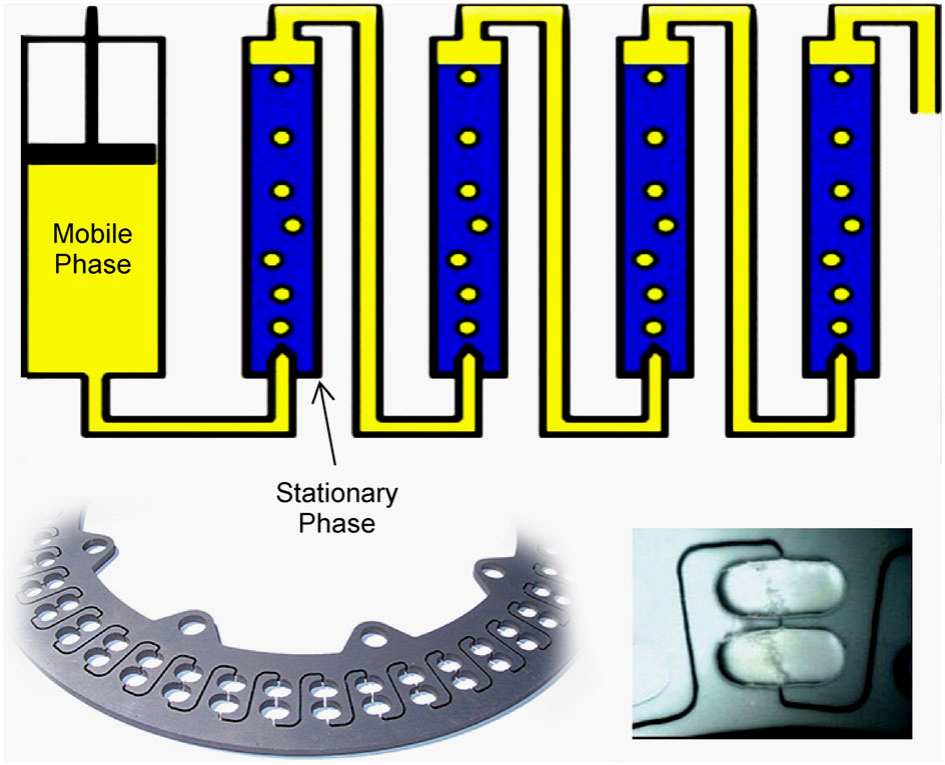
A schematic of a centrifugal partition chromatography (CPC) rotor (*top*) containing a mobile phase (*yellow*) and stationary phase (*blue*) in ascending mode, showing the flow of the light phase (ascending) through the dense phase; a section of a CPC rotor plate showing the chambers (*lower left*) and the chambers in a CPC rotor during operation with a biphasic solvent system (*lower right*)

Since 1970 [22], several variants of CCC have been developed [1]. These include high-performance countercurrent chromatography (HPCCC) [23], high-speed countercurrent chromatography (HSCCC), and centrifugal partition chromatography (CPC). Among the different types of CCC centrifuge systems developed [24, 25], Ito’s J-type centrifuge (Fig. 2) [26] has gained a prominent position due to its suitability for scale-up [27]. In the J-type centrifuge, the coils are connected to the chassis by means of non-rotating flying leads. This is achieved through planetary motion of a coiled pipe, as shown in Fig. 2 [8, 25]. The result is that the head and tail connections of the flying leads flex, but do not exhibit any overall rotation. In the related technique of CPC, a series of linked chambers is rotated around a sun-type axis, and the fluids are pumped through the system of chambers through a pair of rotating seals, as shown in Fig. 3. In general, CPC systems are preferred for larger scale separations (>100 g), and the J-type centrifuge HPCCC systems are used for smaller scale separations (<100 g), although it must be noted that both instrument designs are capable of successfully operating outside these regions.

In HPCCC, also known as hydrodynamic CCC, the stationary phase is retained within a helical coil wound around a drum, while the mobile phase is pumped into one end of the coil. A combination of hydrostatic and hydrodynamic forces is produced as the drum rotates in both planetary and solar motion around a central shaft (Fig. 2). The phases are forced towards opposite ends of the coil [28]; the mobile phase is pumped into the end of the coil, whereas the stationary phase accumulates. The design of the J-type centrifuge [29] produces zones of ballistic mixing and zones of settling as the mobile phase passes through the stationary phase. Compounds carried in the mobile phase elute in order of their distribution ratio (DR) of the compound between the mobile phase and stationary phase. The DR of a compound can be defined as:

1 $$ {\text{DR}} = \, \left[ {\text{SP}} \right]/\left[ {\text{MP}} \right] $$

where [MP] and [SP] are the concentrations of the solute in the MP and SP, respectively [1].

The volumes of the stationary phases used in CCC (and related technologies) enable separations on a much larger scale than is feasible in gas chromatography (GC) or high-performance liquid chromatography (HPLC). Also, in CCC, less solvent is required for a given separation than is required for a similar scale preparative HPLC separation. The performance of the separations in CCC is therefore determined largely by the difference in the partition coefficients of the solutes dissolved in the two liquid phases. It is by careful design of the solvent system that the desired distribution ratios of the solutes can be achieved (see Section 3).

The conventional biphasic solvent systems used in CCC are based on molecular solvents. The most commonly used solvents are mixtures of water, methanol, ethyl ethanoate and hexane [4], and combinations of these are sometimes referred to as the Arizona liquid system [30], or more recently as the “HEMWat” system [31]. Other solvents include butanol, butanenitrile, *N*,*N*-dimethylformamide, dimethyl sulfoxide (DMSO), dichloromethane, ethanenitrile, toluene and trichloromethane, which are known to be both toxic and volatile [32–35]. In addition, it can be difficult to completely remove dipolar aprotic solvents from the isolated product of a CCC separation. The process of solvent selection involves testing the DRs of the solutes being separated, between a large number of solvent systems, and selecting the best overall solvent system. This process can be greatly simplified by the use of automation [6].

## Ionic Liquids in Chromatography

Some of the conventional problems encountered with specialised chromatographic techniques, such as CCC and CPC, originate as a result of the restricted set of solvents which can be utilised as mobile phases. The recent developments in the field of ionic liquids offer opportunities to vastly expand the range of available solvents and provide the ability to tune the solvent properties [21]. In this section, we consider specifically the implications that arise when the application of ionic liquids to chromatography is taken into account.

### Introduction to Ionic Liquids

Ionic liquids were originally defined as salts that are liquid at or below 100 °C [19], composed entirely of ions. More recently, a simpler and more fundamental definition is being used, “an ionic liquid is a liquid containing only ions, derived from a solid which melts before its decomposition temperature”. This removes the artificial imposition of a melting point upon the definition [36].

Ionic liquids have unique properties that can help overcome some of the problems associated with the use of conventional organic solvents (see Section 2). For example, many ionic liquids have no measurable vapour pressure at ambient temperatures and pressures [37], resulting in their non-flammability. In addition, they are usually thermally and chemically stable, which can make them inherently safe and environmentally friendly [19]. By simply varying the anion and cation combination, >10^6^ possible simple ionic liquids, >10^12^ binary mixtures and >10^18^ ternary mixtures can be produced [38, 39]. Ionic liquids can be excellent solvents, being capable of dissolving many inorganic, organic, biochemical and petrochemical materials, sometimes with a remarkably high capacity, thus opening new potential routes for the separation and purification of such compounds, which are difficult to separate by classical methodologies.

The properties of ionic liquids can be adjusted by the choice of the appropriate anion and cation, allowing them to be optimised for specific applications. For example, ionic liquids can be contacted with a second immiscible phase, which could be a molecular solvent, or an aqueous salt solution, or even a second ionic liquid [40]. This gives rise to a huge number of potential biphasic solvent systems that can be optimised for any given separation, generating a wide range of distribution ratios of solutes; the latter in turn can improve separation performance [41, 42]. We term here the use of ionic liquid-based solvent systems, and their use in CCC, as ionic liquid–liquid chromatography (ILLC).

### The Use of Ionic Liquids in Chromatography

The use ionic liquids in chromatography has been growing steadily since 1999, with a total of 2647 publications by the end of 2016, based on a Web of Science search of the terms “ionic liquid” and “chromatography” [18]. As can be seen in Fig. 1, ionic liquids have been found to provide new avenues of investigation in chromatographic science.

In 1999, Anderson and co-workers reported the first use of ionic liquids in GC, utilising their negligible vapour pressure at ambient pressures [37]. These authors used [C_4_mim][PF_6_] or [C_4_mim]Cl as stationary phases coated on the surface of fused silica capillaries. This design allowed the separation of a wide range of organic molecules (alkanes, chloroalkanes, alcohols, amines, aromatics and carboxylic acids) according to their polarity and proton-donor or -acceptor characteristics [20]. Only 7 years later, a full review of applications of ionic liquids in analytical chemistry was published [43].

In 2002, the first use of ionic liquids in capillary electrophoresis was reported to separate Sildenafil and its metabolite UK-103,320 [44]. In the same year, using a stacked disk CPC instrument, a number of salts (referred to as chiral selectors, but could also be described as chiral ionic liquids) were utilised for the enantiomeric separation of *N*-substituted amino acids [45]. More general information on chiral separations using CCC, including those involving ionic chiral selectors, has been reviewed by Ito et al. [46]. It is interesting to note that many of the chiral separations using CCC were carried out on model compounds, which are usually racemic dinitrobenzyl derivatives of amino acids. Very little research exists on the chiral separation of other racemates using ionic liquid-containing solvent systems [47].

In the field of liquid chromatography, the determination of ionic liquid cations by reversed-phase HPLC [48] was reported in 2003, and the first use of ionic liquids as additives to the mobile phase in HPLC was reported in 2004 [49]. The use of ionic liquids as stationary phases in LLC or CCC was detailed by Berthod et al. [16] who measured the partitioning of 38 aromatic compounds in the biphasic ionic liquid solvent system: [C_4_mim][PF_6_]/water. These authors found that “the viscosity of pure RTILs [room-temperature ionic liquids] is too high for direct use as a liquid phase in countercurrent chromatography (CCC)”. A measure of progress in the use of ionic liquids in chromatography is that, for example, Supelco now sells a wide range of capillary GC columns which employ ionic liquids as the stationary phase [50].

Recently, over 40 reviews have been published describing the use of ionic liquids in analytical and chromatographic science, as summarised in Table 1.

**Table 1:** A list of selected reviews published on use of ionic liquids in analytical and chromatographic science

Chromatographic area of research	Year	References
General reviews on the use of ionic liquids in chromatography	2005	[51]
	2005	[52]
	2006	[53]
	2007	[54]
	2007	[55]
	2007	[56]
	2008	[24]
	2008	[57]
	2009	[58]
	2010	[59]
	2011	[60]
	2012	[61]
	2014	[62]
	2016	[63]
The use of ionic liquids in capillary electrophoresis and related technology	2004	[64]
	2006	[65]
	2008	[66]
	2009	[67]
	2013	[68]
	2014	[69]
	2016	[63]
The use of chiral ionic liquids in chromatography	2005	[70]
	2008	[71]
	2008	[72]
	2012	[73]
	2014	[74]
The use of ionic liquids in CCC	2008	[24]
	2013	[75]
The use of ionic liquids in GC	2009	[76]
	2009	[77]
	2011	[78]
	2014	[79]
	2014	[80]
	2016	[81]
	2016	[82]
The use of ionic liquids in HPLC	2006	[83]
	2009	[84]
	2011	[85]
	2012	[86]
	2012	[87]
	2015	[88]
Chromatographic measurement of ionic liquid properties	2004	[89]
The use of ionic liquids in liquid chromatography	2007	[90]
	2014	[91]
	2015	[92]
	2015	[93]
The use of ionic liquids in micellar chromatography	2012	[94]
The use of ionic liquids in supercritical fluid chromatography	2009	[95]
The use of ionic liquids in hydrophilic interaction chromatography	2016	[96]

### The Use of Ionic Liquids in Countercurrent Chromatography

The aim of the research into ILLC is to develop a technology that is capable of separating mixtures of most soluble organic compounds [97], with a similar goal for inorganic compounds [98], using the methodologies based on the principles of CCC [21]. Due to the very large number of ionic liquids that can be used as stationary phases and mobile phases [99], a wide range of separations should be achievable. With more than one million possible ionic liquids [19, 38], the number of possible phases that can be generated is immense. In conventional LLC or CCC, only about a dozen different molecular solvents are commonly used, and these are usually based on mixtures of three to five (or more) solvents in order to generate useable biphasic solvent systems [100].

Berthod et al. carried out the initial testing of diluted ionic liquids in CCC instruments between 2003 and 2009 [16, 24, 101, 102]. This research group noted and reported that the use of ionic liquids in CCC had a number of drawbacks, such as “UV absorbance limiting the use of the convenient UV detector”, “non-volatility precluding the use of the evaporative light-scattering detector for continuous detection” and “high viscosity producing pressure build-up” [101]. Berthod et al. went on to conclude that CCC was unsuitable at that time, as the instrumentation available “was not able to cope with the high back-pressures generated by the large viscosities of many ionic liquids” [101].

The UV–Vis absorbance of ionic liquids can be problematic and interfere with detection of the analyte. The absorbance depends on the structure of the ionic liquid being used, and the absorbance of both the anion and cation must be separately taken into account. For example, imidazolium cation ionic liquids absorb light strongly at wavelengths below 260 nm. However, ammonium and phosphonium cation ionic liquids can be used over the whole 200- to 800-nm range of modern UV–Vis spectrophotometers. Anions (such as chloride) can also be used over the full 200- to 800-nm range, but anions such as triflate or bis{(trifluoromethyl)sulfonyl}amide absorb light at wavelengths below 250 nm. Coloured impurities in the ionic liquid can also affect the performance of the UV–Vis detector, but these can be removed by filtering the ionic liquid through charcoal [103]. A second problem with UV–Vis detectors is that small bubbles of stationary phase can be present in the mobile phase as it elutes from the coil, and these bubbles produce spikes in the chromatogram. Careful orientation or design of the UV–Vis flow cell can help minimise this problem.

The non-volatility of ionic liquids can be problematic for the use of evaporative light scattering detectors (ELSDs) [101]. Provided that the mobile phase contains very low concentrations of ionic liquids, as is the case with many solvent systems, this detector may be used, but factors such as unintended bleeding of the stationary phase into the mobile phase eluting from the coil make the use of ESLDs unviable. Alternatively, two types of detectors that could be used are refractive index and conductivity detectors, although none has been tested at the time of writing.

To overcome the greater viscosity of ionic liquid solvent systems, the CCC instrumentation requires careful design and construction of the fluid flow paths to prevent excessive pressure build-up, particularly at high flow rates. For ILLC, the machine, based on a J-type centrifuge, must withstand high working pressures (at least 50 bar) for extended periods of time. This requires that the coils be made from either stainless steel or, in the case of highly corrosive materials, titanium. The centrifuge must also be capable of running at elevated temperatures (up to 50 °C), which, through a reduction of viscosity, can dramatically improve the ILLC separation performance [21, 97, 98, 104]. Based on these requirements, AECS-QuikPrep Ltd. (London, UK) produce CCC instruments suitable for use with ionic liquid solvent systems [104].

It is not usually possible to use a neat ionic liquid in CCC because the addition of a second immiscible solvent often dilutes the ionic liquid as a result of mutual solubility effects described in Section 3.4 [105]. Berthod et al. [101] explicitly state: “Mutual solubility with other solvents is a critical issue, because the viscosity of [room-temperature ionic liquids] is too high to be of practical use in CCC”. For example, the ionic liquid [C_4_mim][PF_6_] has a viscosity of 312 cP [16, 106] when pure, which is far too high to be reliably pumped by an HPLC pump. Berthod et al. found that the phase [C_4_mim][PF_6_]/water (1.8% v/v water) had a viscosity of approximately 30–40 cP at 20 °C, but this was “too viscous, for easy CCC use” [16, 106]. The addition of ethanenitrile (20–40% w/w) to the [C_4_mim][PF_6_]/water solvent system was required to obtain successful operation of the CCC instrument [101]. Unfortunately, also in 2003, Rogers and coworkers [107] found that the ionic liquid [C_4_mim][PF_6_] can undergo hydrolysis. Thus, the [PF_6_]^−^ anion can hydrolyse to give a mixture of aqueous phosphate (or phosphoric acid) and hydrofluoric acid, which is highly toxic and corrosive [107]. This problem is solved by using the bis{(trifluoromethyl)sulfonyl}amide [NTf_2_]^−^ anion as a direct replacement for the [PF_6_]^−^ anion. Not only is the [NTf_2_]^−^ anion hydrolytically stable, but ionic liquids derived from it tend to have low viscosities [108].

For the separation of polar and/or strongly hydrogen bonding molecules, such as saccharides or proteins, aqueous biphasic solvents (ABS) [109] have been successfully used in CCC [110, 111]. These are usually based on concentrated aqueous inorganic salt solutions, combined with a second aqueous phase, saturated with an organic solvent [such as ethanol or polyethylene glycol (PEG)] [112, 113], which employ the mismatch between kosmotropic and chaotropic interactions (increasing or decreasing, respectively, the structuring of water) to produce biphasic solvent systems [114, 115]. For example, they have been used in the CPC separation of saccharides using the ethanol/DMSO/aqueous ammonium sulfate (300 g l^−1^) (0.8:0.1:1.8, v/v/v) solvent system [116]. Berthod et al. found that chaotropic chloride ionic liquids (such as [C_4_mim]Cl) are immiscible with many kosmotropic concentrated salt solutions and that these solvent systems can be used in a CCC machine [117]. The ABS system [C_4_mim]Cl/aqueous 2.5 M K_2_[HPO_4_], first described by Berthod et al., has been used for the purification of the anti-cancer drug lentinan [42]. Here, the ionic liquid-based ABS solvent system [C_4_mim]Cl/aqueous 2.5 M K_2_[HPO_4_] has a much greater solute capacity (approx. tenfold greater) than the previously used PEG-based ABS solvent system (PEG 1000/K_2_[HPO_4_]/K[H_2_PO_4_]/water (0.5:1.25:1.25:7.0, mol/mol/mol)) [113]. The ionic liquid ABS solvent system allows for much larger amount of lentinan to be separated by boosting the space–time yield of the chromatographic apparatus. Finally, a related approach for separating polar compounds in CCC involves the use of deep eutectic solvent systems (such as mixtures of choline chloride/glucose) [118].

Since the initial work of Berthod et al. in ILLC, there have been a number of papers describing the use of ionic liquids in CCC. These include the analysis of mycotoxins [119] and chlorophenols in red wines [120], the potential use of aqueous biphasic solvent systems containing ionic liquids in the separation of caffeine from xanthine and nicotine [121] and in the separation of neomangiferin and mangiferin from* Rhizoma anemarrhenae*, all using the hydrolytically unstable [C_4_mim][PF_6_] [107] as an additive to aqueous ethyl ethanoate [122].

### Phase Behaviour of Ionic Liquids

**Fig. 4:**
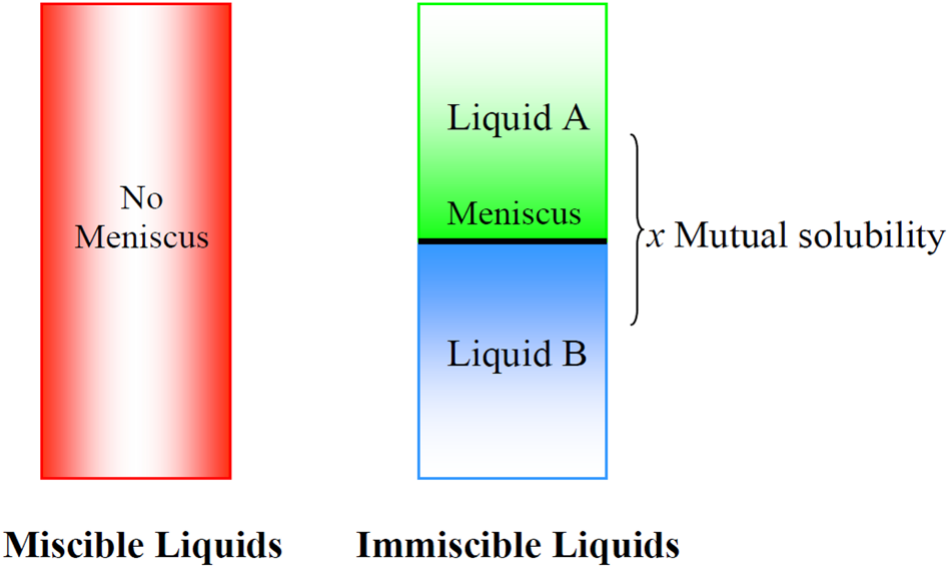
The effect of mixing two immiscible liquids. Each liquid phase is partially soluble in the opposite immiscible liquid phase

Two immiscible liquids are present when there is a visible barrier between two phases. However, immiscibility is not the same as insolubility, as shown in Fig. 4. For the purpose of CCC separations, the phases chosen (either ionic liquid–organic solvent, ionic liquid–water, or mixtures of ionic liquids) should be immiscible, but they will possess a degree of solubility in each other.

Ionic liquid-molecular solvent biphasic systems are very useful in chromatography, as they allow for easy analyte isolation and solvent recovery. There is usually very little ionic liquid contained in the molecular solvent phase, and so the molecular solvent usually is employed as the mobile phase. Solvent recovery usually involves distillation of the volatile solvent from the ionic liquid phase and the analyte-containing mobile phase, which can then be directly reused [123]. Also, ionic liquid solvent systems usually contain just two or three components rather that four or five, as is the case with molecular solvents. This makes solvent recovery much more straightforward [6] due to the lower number of volatile components that need separating. Solutes that remain dissolved in an ionic liquid phase after the volatile solvent recovery step can be removed by a number of techniques, such as solvent extraction, precipitation with an antisolvent or filtration of a solution of the ionic liquid through silica or charcoal, or a combination of these methods [103].

#### Ionic Liquid-Organic Solvent

The miscibility of ionic liquid–organic solvent mixtures is an important property in chromatography and influences the analyte distribution ratios. Many solvents can form biphasic mixtures with ionic liquids, where the solvent is soluble in the ionic liquid phase, whereas the ionic liquid is poorly soluble in the solvent phase. Eight examples of the mutual solubilities of ionic liquids with an organic solvent (hexane) are shown in Table 2. The eight ionic liquids tested were [cholinium][NTf_2_], [C_*n*_py][NTf_2_] (*n* = 2, py = pyridinium), [C_*n*_mim][NTf_2_] (*n* = 2, 4, 6, 10, or 12) and [P_6 6 6 14_][NTf_2_]. The ionic liquid phases contain significant amounts of hexane, but the organic phase (hexane) does not contain the ionic liquid [124, 125].

**Table 2:** Composition of the biphasic ionic liquid/hexane solvent systems, showing the solubility of hexane in the ionic phase [124, 125] at 25 °C

Ionic liquid^a^	Ionic phase composition (mol%)		Organic phase composition/mol%
	Ionic liquid	Hexane	Hexane
[cholinium][NTf_2_]	98.5	1.5	>99.9
[C_*n*_py][NTf_2_]	95	5	>99.9
[C_2_mim][NTf_2_]	96	4	>99.9
[C_4_mim][NTf_2_]	91	9	>99.9
[C_8_mim][NTf_2_]	70	30	>99.9
[C_10_mim][NTf_2_]	58	42	>99.9
[C_12_mim][NTf_2_]	44 (79, 87)^b^	56 (21, 13)^b^	>99.9
[P_6 6 6 14_][NTf_2_]	9 (30, 37)^b^	91 (70, 63)^b^	>99.0

^a^See Abbreviation List for details

^b^Ionic phase composition is given in mol%, followed in parenthesis by vol% and wt%, respectively

The effect of dissolving a molecular solvent in an ionic liquid dramatically reduces the ionic phase’s viscosity [105, 126] to the point that it can effectively be pumped with an HPLC pump. This has enabled viscous hydrophobic ionic liquids, such as [P_6 6 6 14_]Cl (viscosity = 2729.1 cP at 25 °C), to be used in separations of metal salts, as discussed later in this chapter [127].

An important factor to be considered in the selection of ionic liquids used in CCC is the rate of exchange of solutes between the two liquid phases, which depends in part on the viscosity of the phases used in CCC. This experimental observation is valid irrespective of the nature of the two phases employed. The higher viscosities of ionic liquid phases can reduce the equilibration rate of the solutes between the two liquid phases and can result in the problem of band-spreading [128]. This can be partially offset by running the separation at higher temperatures or by diluting the ionic liquid phase. However, the viscosities of ionic liquids are highly dependent on the nature of both the anion and the cation. One approach to reducing the viscosity of the ionic phase is to use fluorinated anions, such as [OTf]^−^ (trifluoromethanesulfonate) or [SO_3_CF_3_] or [NTf_2_]^−^. Also, using cations containing asymmetrical, delocalised charge centres can lower the viscosity.

**Fig. 5:**
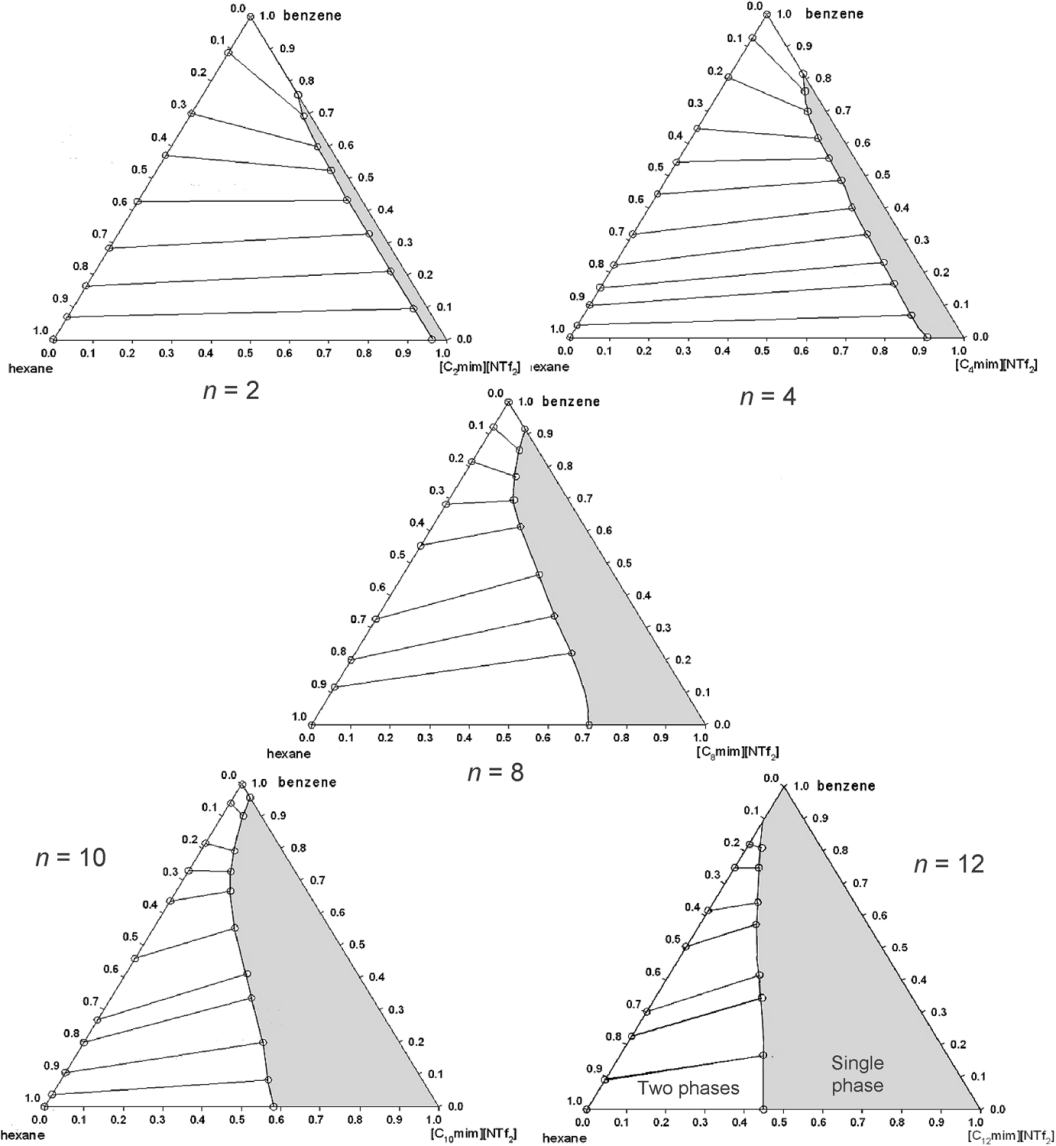
The effect of the variation in the length of the alkyl chain of 1-alkyl-3-methylimidazolium bis{(trifluoromethyl)sulfonyl}amide [*C*_*n*_ *mim*][*NTf*_*2*_], where* n* = 2, 4, 8, 10 or 12, on the phase equilibria of benzene dissolved in a hexane/[C_*n*_mim][NTf_2_] ionic liquid solvent system [124]

The chemical structure and spatial charge distribution of ionic liquids can be varied in order to alter their phase behaviour in biphasic solvent systems. An example is the solubility and distribution ratio of benzene in the hexane-[C_*n*_mim][NTf_2_] (*n* = 2, 4, 6, 10 or 12) biphasic solvent systems [124] (Fig. 5). The solubility of benzene increases from 23 mol% in the [C_2_mim][NTf_2_] phase to complete miscibility with [C_12_mim][NTf_2_]. The three-phase diagrams clearly show the dramatic effect of how changes in the cation give rise to very large variations in the phase behaviour. Also, the ionic liquids used are not measurably soluble in the hexane phase. This property can be used to completely separate benzene from hexane, using a hexane mobile phase in liquid–liquid extractions [124, 125].

**Fig. 6:**
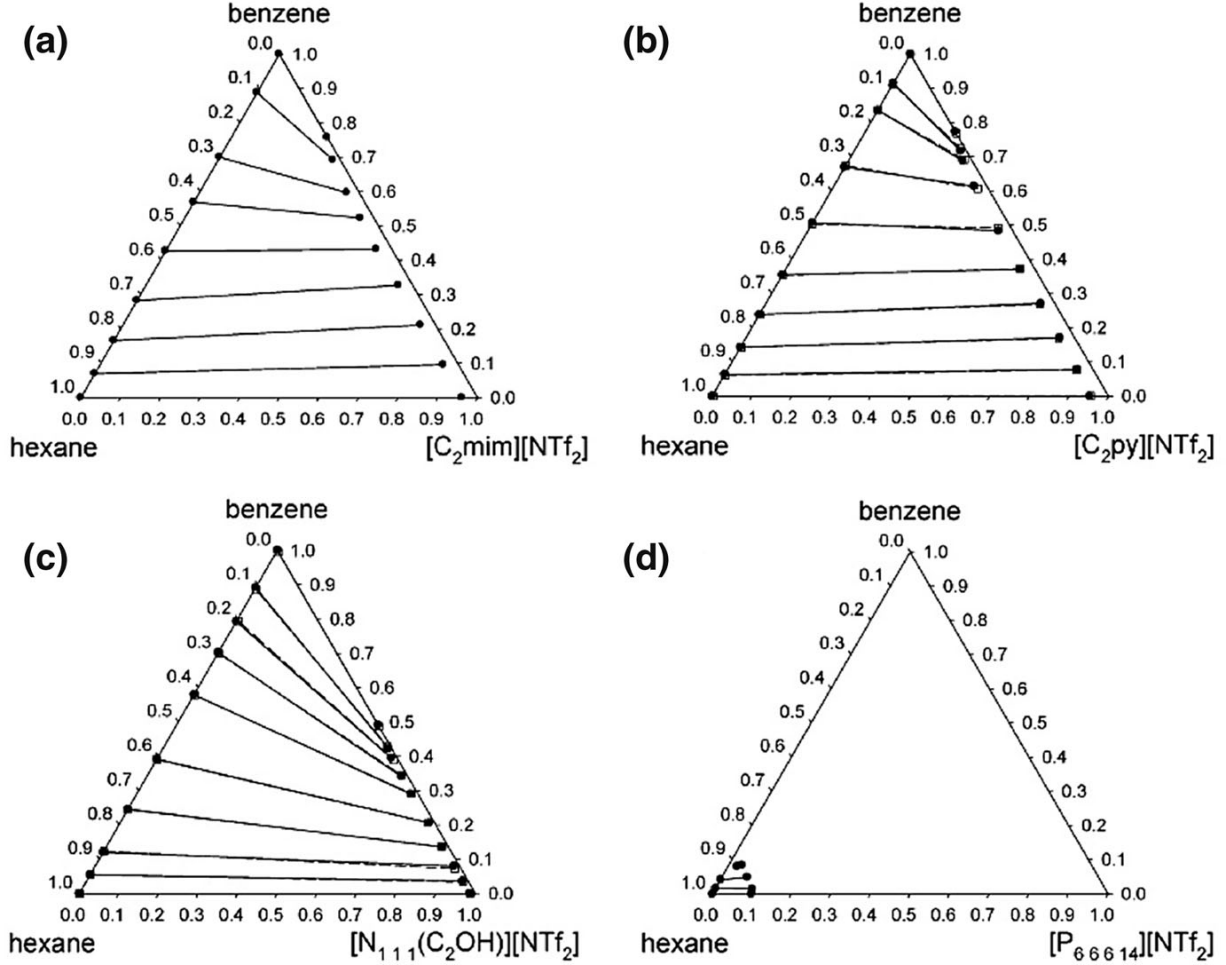
The effect of the cation on the phase equilibria of benzene dissolved in a hexane/[cation][NTf_2_] ionic liquid solvent system [125]. Cation: **a** 1-ethyl-3-methylimidazolium ([C_*2*_mim]^+^), **b** 1-ethylpyridinium ([C_2_py]^+^), **c** (2-hydroxyethyl)trimethylammonium, **d** trihexyltetradecylphosphonium

The type of the cation used in four [NTf_2_]^−^ anion ionic liquids has also been shown to affect the distribution ratio of benzene [125] and, more generally, aromatic compounds [129]. In Fig. 6, the biphasic hexane/[cation][NTf_2_] solvent systems (where cation = [C_2_mim]^+^, [C_2_py]^+^, (2-hydroxyethyl)trimethylammonium, or [P_6 6 6 14_]^+^) show a remarkable difference in their phase behaviour, with the solute distribution ratios of benzene in the solvent systems at 40 °C being 1.20, 1.24, 0.69, and 0.94, respectively [125]. This information is useful in ionic liquid–liquid chromatography and demonstrates how the design of an ionic liquid affects the distribution ratios of solutes. A number of reviews [115, 130–133] and further information on the liquid–liquid equilibria of ionic liquids have been published elsewhere.

#### Ionic Liquid–Water Systems

Mixtures composed of an organic polymer, such as PEG, and an inorganic salt, such as sodium or potassium hydrogen phosphate (K_2_[HPO_4_]), are able to separate into two layers, as first described by Albertson [134]. Such mixtures are known as aqueous biphasic systems, as both layers are aqueous solutions of different compositions: the upper layer contains most of the PEG, whereas the lower layer contains most of the inorganic salt. Aqueous biphasic systems have proven to be useful in CCC for the purification of biological materials [121, 135–137].

Using ternary mass or molar phase diagrams, Berthod and coworkers studied the putative formation of ionic liquid-based aqueous biphasic systems by mixing [C_4_mim]Cl with aqueous solutions of inorganic salts (K_2_[HPO_4_], K_3_[PO_4_], Na_2_[HPO_4_], Na[H_2_PO_4_], Na_2_[CO_3_] or bases (NaOH and KOH) [21, 22, 24]. In addition, these researchers found out that the [C_4_mim]Cl/K_2_[HPO_4_]/water system was much easier to retain in the CCC columns than the similar aqueous biphasic system containing PEG 1000. However, the significant polarity difference between PEG 1000 and [C_4_mim]Cl aqueous biphasic systems is responsible for the different behaviour of the two selected systems towards protein separation using CCC variants.

There have been a number of other studies on the phase behavior of ionic liquids in aqueous biphasic solvent systems. These include the study on [C_4_py]Br/H_2_O/Na_2_SO_4_ at various temperatures by Li and coworkers [138] and that on choline-amino acid ionic liquids/K_2_CO_3_/H_2_O by Li and coworkers [139], as well as the analysis on an extensive range of these ionic liquid-based aqueous biphasic solvent systems by Sen and Chakraborty [140].

As hydrophobic ionic liquids can easily form biphasic systems with water, they are possible alternatives in CCC to the existing [C_4_mim]Cl/inorganic salt aqueous biphasic systems. Most hydrophobic ionic liquids are very poorly soluble in water phases, but water has appreciable solubility in the ionic liquid phase. Thus, if the mobile phase in CCC is water, then product isolation is simply a matter of evaporating the water phase [40, 124, 125, 129, 141–144].

#### Ionic Liquid–Ionic Liquid

**Fig. 7:**
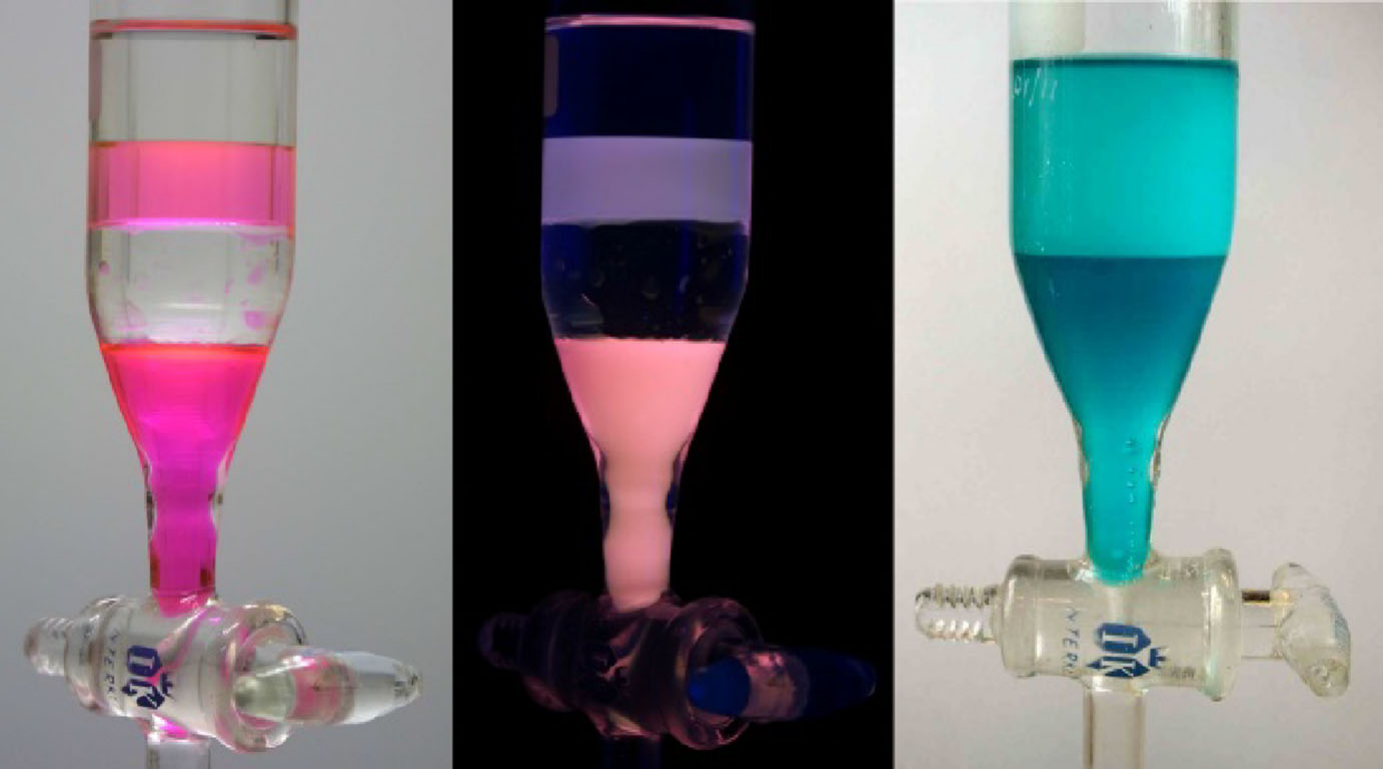
A picture of a four-phase solvent system (*left* and *centre*) composed of (*top* to *bottom*) hexane, trihexyltetradecylphosphonium bis{(trifluoromethyl)sulfonyl}amide ([P_6 6 6 14_][NTf_2_]), water and 1-ethyl-3-methylimidazium bis{(trifluoromethyl)sulfonyl}amide ([C_2_mim][NTf_2_]), using eosin Y as a luminescent dye which is soluble in the two ionic liquid-containing phases. The central photograph is of a system illuminated with 254-nm UV light. Also shown is a two-phase solvent system (*right*) of [P_6 6 6 14_]Cl (*upper* phase) and [C_2_mim] Cl (*lower* phase) with cobalt(II) Cl as the blue chromophore. In the two blue phases, the complex is tetracobaltate(II) [40]

The first examples of ionic liquids which were shown to be mutually immiscible with each other were published in 2006 [40]. Similarly, tetraphasic solvent systems can be constructed, as shown in Fig. 7 [40]. Mutually immiscible ionic liquid systems can be produced by mixing very hydrophilic ionic liquids with very hydrophobic ones, such as with [P_6 6 6 14_]Cl and [C_2_mim]Cl (Fig. 7 right image).

**Fig. 8:**
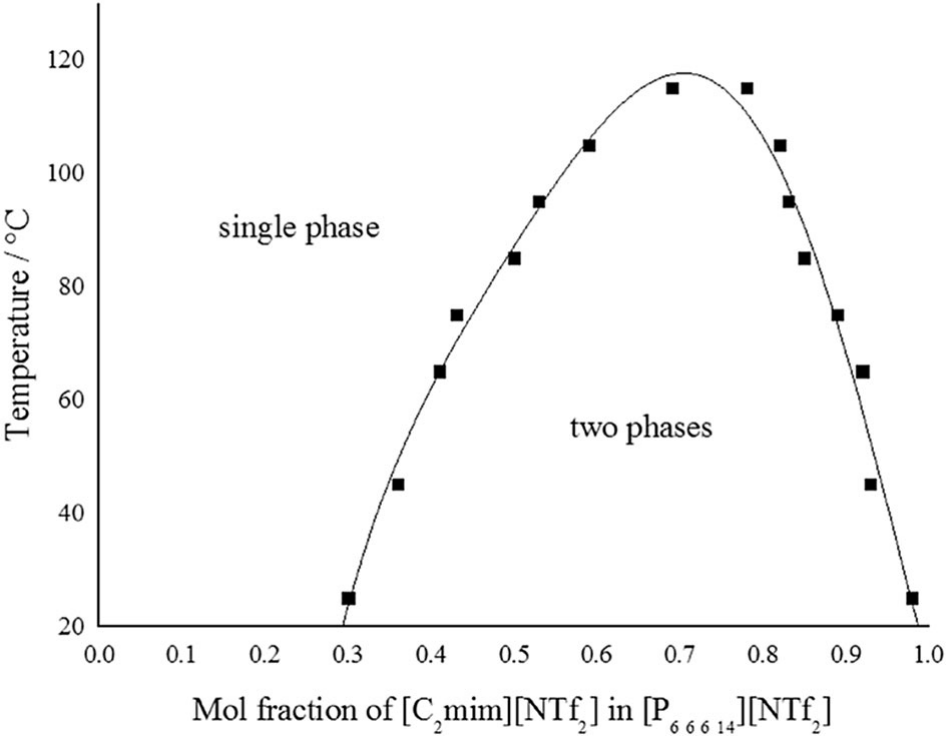
The phase diagram of two ionic liquids at varying temperatures [40, 141]

**Fig. 9:**
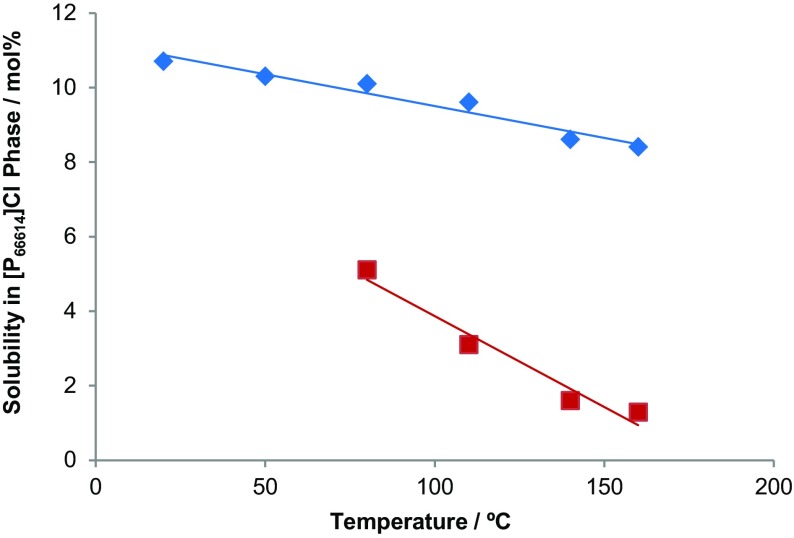
The solubility of [C_1_mim]Cl (*red line*) and [C_2_mim]Cl (*blue line*) in the immiscible [P_6 6 6 14_]Cl phase at various temperatures [40]

**Fig. 10:**
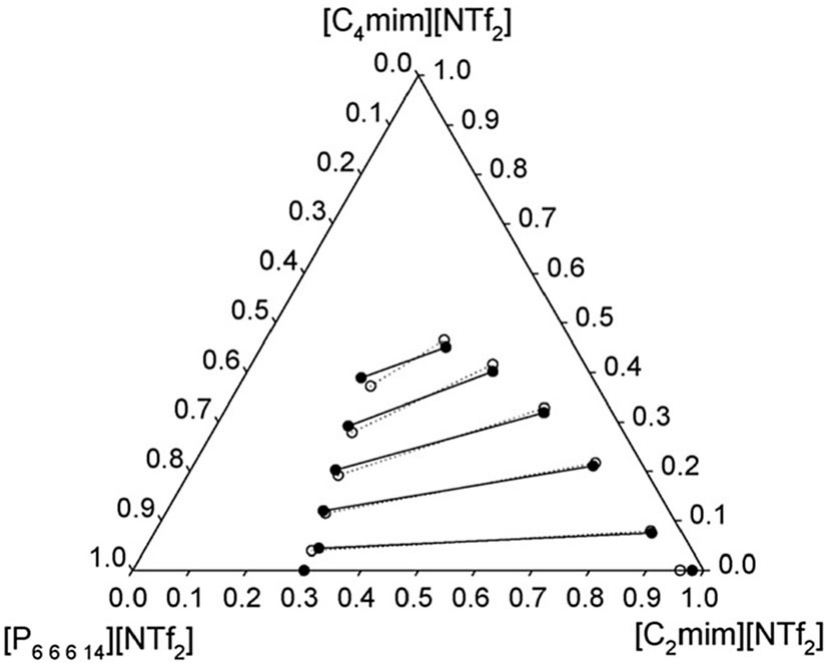
The ternary phase diagram of a mixture of three ionic liquids: [C_2_mim][NTf_2_], [C_4_mim][NTf_2_], and [P_6 6 6 14_][NTf_2_] at 25 °C (*solid circle*) and 40 °C (*open circle*) [141]

The mutual miscibility of two ionic liquids, namely [C_2_mim][NTf_2_] and [P_6 6 6 14_][NTf_2_], at different temperatures is shown in Fig. 8 [40]. This biphasic solvent system becomes completely miscible as the temperature rises. In contrast, in the cases of [C_*n*_mim]Cl (*n* = 1 or 2)/[P_6 6 6 14_]Cl, the mutual solubility decreases with increasing temperature (see Fig. 9) [40]. It is also possible to construct a ternary phase diagram for three ionic liquids, as shown in Fig. 10.

### Solvent Engineering and Ionic Liquid–Liquid Chromatography

The inherent tunability of ionic liquids imparts control over their physicochemical properties, such as viscosity, hydrophobicity, hydrophilicity, density, surface and interfacial tensions and corrosivity. Moreover, and of vital importance, the distribution ratios of a solute between an ionic liquid phase and a second phase (which may be either water, an organic solvent or another ionic liquid) can be controlled by the design of the ionic liquid. For example, if a solute in a biphasic hexane–ionic liquid solvent system is not soluble enough in the ionic liquid phase, then the solvent engineering approach is to redesign the ionic liquid cation to have longer alkyl chains. Alternatively, the anion can be redesigned and replaced with a larger (usually with longer alkyl chains) and/or the use of a more charge delocalised anion. As will be demonstrated later, this versatility allows a general purpose methodology for a very large number of separations to be developed, including separations that were previously thought to be too difficult to implement.

## Ionic Liquid Solvent Systems and Instrumentation in Countercurrent Chromatography

### Introduction

In order to carry out CCC with ionic liquids, the instrument must be designed to cope with much higher backpressures than those that are encountered with many molecular solvents. In addition, the viscosity of ionic liquids in a CCC machine has received little attention in the literature. In this section, the instrument design, the solvent systems and their viscosities are described.

### Instrument Design

The Quattro IL-Prep™ ILLC instrument AECS-QuikPrep Ltd, London, UK contains two pairs of stainless steel tubing coils mounted on each of the two weight- and dynamically matched bobbins. The coils are held in place by aircraft-quality adhesive. Stainless steel is used for the coil tubing rather than the more conventional PTFE tubing [145, 146]. This change was implemented in order to allow the instrument to operate at up to 70 bar backpressure.

**Fig. 11:**
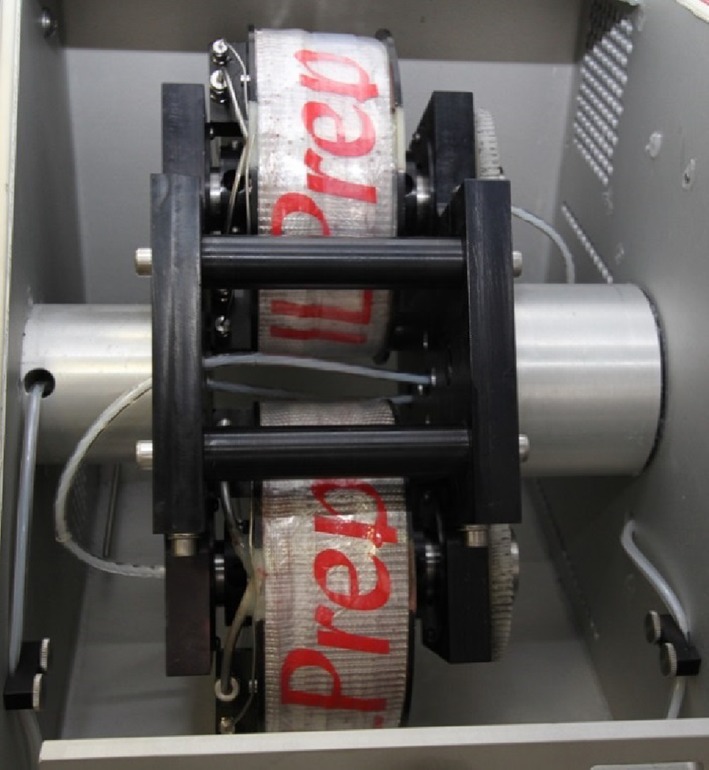
The internal view of the four coils in the planetary centrifuge of the Quattro IL-Prep™ ILLC instrument.* ILLC* Ionic liquid–liquid chromatography

The coils (see Fig. 11) are connected to the pumps and detector using poly(1,1,2,2-tetrafluoroethylene) (PTFE) flying leads with an internal diameter (ID) of 0.8 mm (coils 1 and 2) and 0.5 mm (coils 3 and 4) and an external diameter (ED) of 1.6 mm (all coils). It was found that the 0.5-mm ID tubing had a greater service lifetime. The sample injection loops range in size from 0.5 to 10 cm^3^ and are made from 0.8-mm ID stainless steel or 1.6-mm ID PTFE tubing. Further details and dimensions of the four coils are given in Table 3.

**Table 3:** Details of the J-type centrifuge coils installed within the IL-Prep ILLC instrument

Coil number	Bore ID (mm)	Length (L) (m)	Number of turns	Capacity (V_c_/cm^3^)	L/ID ratio	Flow velocity (mm s^−1^)^a^
1	1.0	13.35	26	12	13,350	12.73
2	2.1	36.02	76	133	17,152	2.887
3	1.0	39.89	78	34	39,890	12.73
4	3.7	23.83	52	236	6441	0.930

ID, internal diameter

^a^The flow velocity is defined as the speed of movement of a single phase of fluid along the length of the coil at an applied fluid flow rate of 1.0 cm^3^ min^−1^

The J-type multilayer coil planet centrifuge (where the coils are rotated in planetary motion around a central axis) [145] has a revolution radius of 13 cm and produces a synchronous planetary motion of the coil holder. The *β*-value (the ratio of the planetary radius to the ratio of sun radius) of the pipes in the coil varies from 0.60 at the internal layer to 0.90 at the external layer [25]. The IL-Prep system is equipped with two HPLC pumps (0–40 cm^3^ min^−1^), a UV–Vis detector with four simultaneous channels (190–800 nm), a data station and a fraction collector. The rotation speed of the IL-Prep™ instrument can be varied between 0 and 865 rpm, and the temperature of the coils can be controlled between 20 and 50 °C. The instrument, pumps and tubing can be operated at up to 70 bar pressure, and it has been tested by running for 48 h continuously at the maximum operating pressure, temperature and rotor speed [104].

**Fig. 12:**
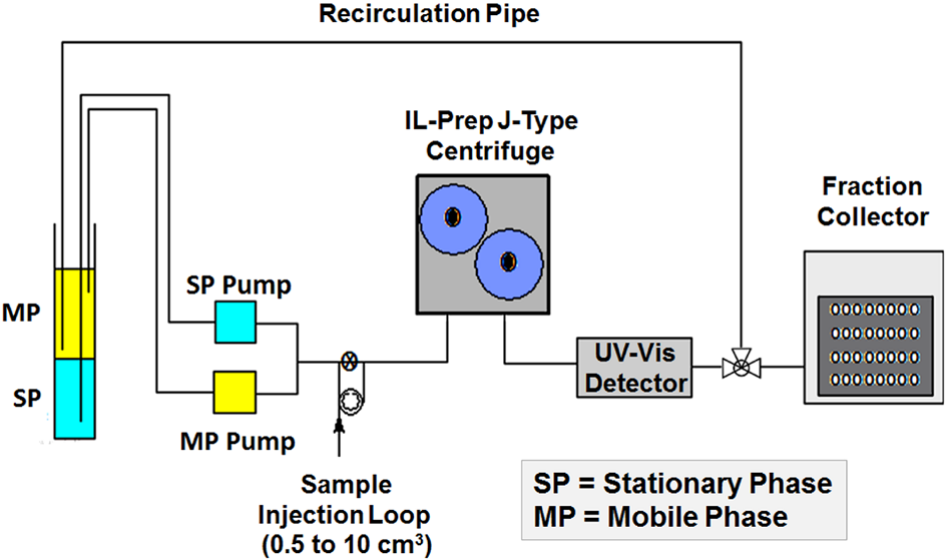
The configuration of the IL-Prep™ instrument

The ILPrep™ instrument can be operated in two modes. In dense phase as the stationary phase (DPS) mode, the mobile phase is pumped from the tail to the head of the coil; in light phase as the stationary phase (LPS) mode, the mobile phase is pumped from the head to the tail of the coil. The configuration is represented schematically in Fig. 12. The two immiscible phases are held in a large measuring cylinder, the two phases being pumped separately into the coils. When the solvent phases are not being collected, they are returned to the measuring cylinder via a return or recirculation pipe. This arrangement allows the contents of the coil to be calculated from the variation in the levels of the two phases in the measuring cylinder. Also, to assist in the calculation of the phase contents of the coil, the dead volume of the piping must be known (in the present case 4.0).

### Solvent Selection

**Fig. 13:**
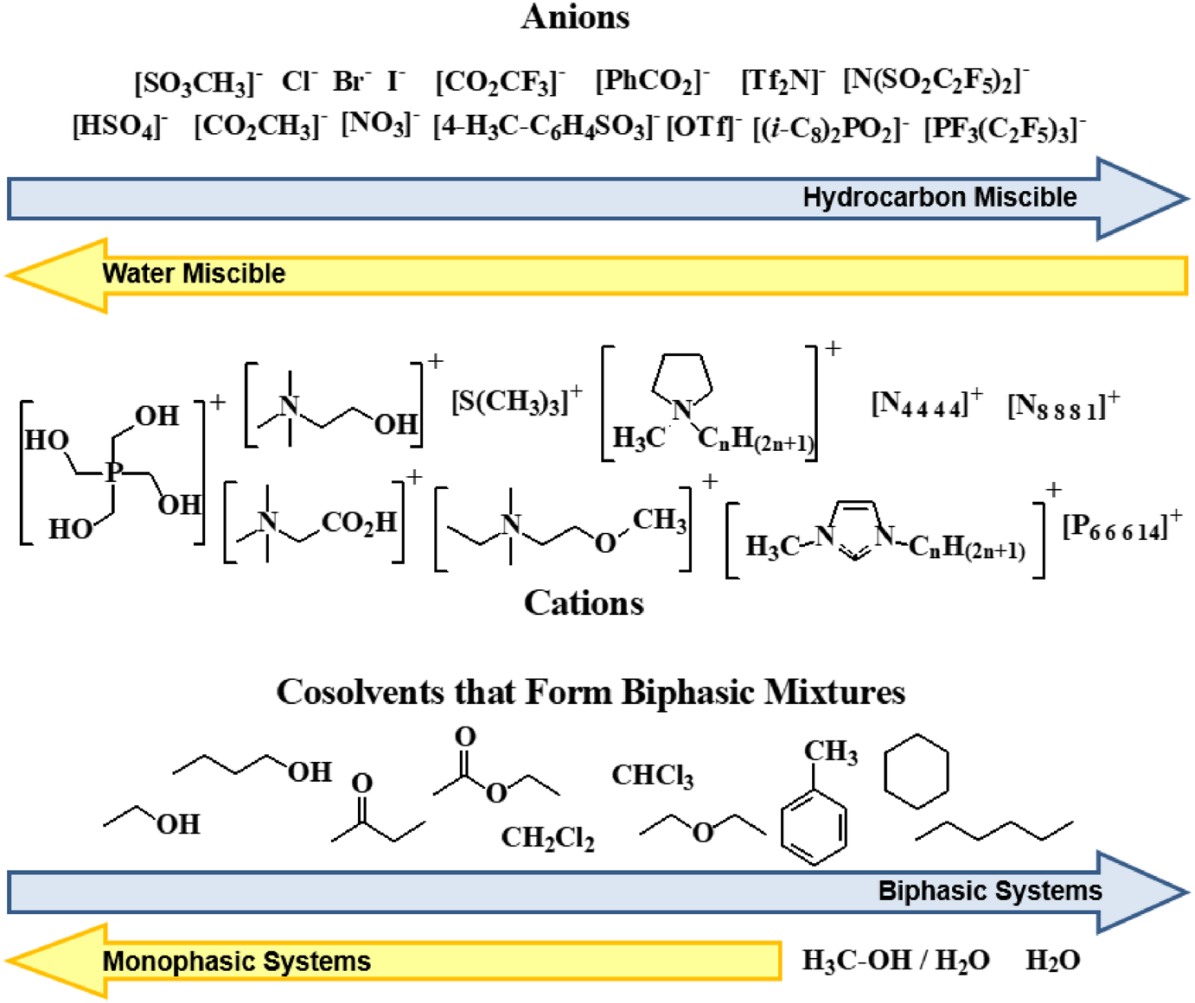
Qualitative water and hydrocarbon miscibility of commonly used ionic liquid-forming anions and cations. Ionic liquids made from ions on the *left* (on both the *upper* and *lower rows*) are hydrophilic and more soluble in polar solvents, and ionic liquids made from ions on the *right* are more hydrophobic and hydrocarbon soluble. In order to form two-phase solvent systems for use in ionic liquid–liquid chromatography (ILLC), solvents in the *lower right* part of this figure are more likely to form biphasic mixtures

There is a huge number of possible ionic liquid phases, and their selection must be made by considering their reactivity (or, perhaps, their lack of it), cost, viscosity, density, solvent behaviour and interfacial tension, among others. This requires both experience and a database of ionic liquid properties [147–149]. For the separations discussed here (Table 4), the biphasic combinations of ionic liquids and molecular solvents were chosen based on previous research into ionic liquid phase behaviour and solvent extraction studies (also see Section 3.4) [40, 42, 124, 125, 129, 141–143, 149]. One of the simple ways to find and select biphasic system consisting of ionic liquids and other solvents is demonstrated in Fig. 13. Here, a qualitative water and organic solvent miscibility guide for commonly used ionic liquid forming anions and cations is shown. In general, ionic liquids made from anions and cations on the left are hydrophilic and more soluble in polar solvents. Ionic liquids made from anions and cations on the right of the chart shown in Fig. 13 are generally hydrophobic and soluble in most organic solvents, except some alkanes; ionic liquids made from ions on the left of the chart are immiscible with most organic solvents less polar than butanol. Another way of looking at this (in the lower part of Fig. 13) is that hydrocarbons are immiscible with most ionic liquids, but solvents like butanol are only immiscible with ionic liquids on the far left of the chart [150]. The hydrophobicity, or hydrophilicity, of ionic liquids can also be calculated using COSMO-RS software [149].

**Table 4:** Phase combinations used for ionic liquid–liquid chromatography separations

Stationary phase^a^	Mobile phase	Separations	Mode
[C_12_mim][NTf_2_] (A)	Hexane	Alkene/oxygenated sesquiterpenes	DPS
[C_10_mim][OTf] (B)	Hexane	Alkane/aromatics	DPS
[C_4_mim]Cl (C)	3.0 M K_2_[HPO_4_]	Glucose/sucrose	LPS
[P_6 6 6 14_]Cl/ethyl ethanoate (D)	Water	Co(II), Ni(II), Cu(II) dichlorides	LPS
3.0 M K_2_[HPO_4_] (E)	[C_4_mim]Cl	Lentinan	DPS

DPS, Dense phase stationary (in biphasic solvent systems); LPS, light phase stationary (in biphasic solvent systems)

^a^Uppercase letters A-E in parenthesis are also used in Table 5, to refer to the solvent system composition. See Abbreviation List for details

It is a requirement that the combination of ionic liquids and molecular solvents used in ILLC forms a biphasic mixture and that there is a significant density difference between these two immiscible phases (preferably >0.10 g cm^−3^). For example, [C_10_mim][OTf] cannot be used effectively in combination with water because the two phases (water and water saturated ionic liquid) have similar densities and do not separate from each other easily. However, [C_6_mim][OTf] and [C_8_mim][OTf] can be used in combination with water because the ionic liquids are significantly more dense than water. A second factor to consider in ILLC solvent selection is viscosity. An extensive list of ionic liquid viscosities can be found in the literature [41], but these data can only be used as a rough guide to determine which ionic liquids are suitable. As mentioned earlier in this chapter, the viscosity of the ionic phase in a biphasic mixture of a molecular liquid with an ionic liquid is dramatically reduced [105]. This reduction in viscosity of the ionic phase can be estimated using Eyring’s absolute rate theory [151].

Ionic liquid selection involves finding or synthesising an ionic liquid that does not irreversibly react with the solutes being separated, but does have a high enough solubility for those solutes. Generally, an ionic liquid with coordinating anions, such as halides, short chain carboxylates or short chain sulfonates, are selected for polar or strongly hydrogen-bonding solutes. Ionic liquids with weakly coordinating anions, such as [OTf]^−^ or [NTf_2_]^−^, are selected for solutes of low or no polarity. Next, an ionic liquid-immiscible solvent (usually water for polar solutes or a hydrocarbon for non-polar solutes), which also exhibits high solute solubility, is selected. The distribution ratios of the solutes between the two immiscible phases must then be measured (e.g. by electronic absorption or nuclear magnetic resonance spectroscopy). Finally, the ionic liquid is either redesigned, or a co-solvent is added to both phases, to adjust the distribution ratios so that they lie in the range of 0.25–4.0. It should be noted that addition of a co-solvent (or second molecular solvent) to an ionic liquid–molecular solvent biphasic mixture will significantly affect the distribution ratios of the solutes, as well as significantly reduce the viscosity of the mixture [101]. This is a very effective way of fine-tuning the solute distribution ratios. Finally, a choice is made about which phase is to be used as the mobile phase and stationary phase. Generally, the mobile phase that is chosen is that from which it is easiest to remove the solute. Normally this means that the ionic liquid should be used as the stationary phase, as in Table 4 (Stationary phase label A–D). One exception to this is found in Table 4 (Stationary phase label E), where an ionic liquid was chosen as the mobile phase, since the solute (lentinan) can be precipitated from the ionic phase simply by adding ethanol.

Finally, some ionic liquid–molecular solvent combinations should be avoided due to the formation of unbreakable emulsions. Ionic liquids can in some cases be used to break (demulsify) emulsions [152], but surface active ionic liquids like [C_12_mim]Cl [153], especially when combined with halogenated solvents (CH_2_Cl_2_ or CHCl_3_), can produce emulsions which do not separate on the timescales encountered in CCC.

### Stationary Phase Retention Curves with Ionic Liquids

Obtaining a stationary phase retention curve is a prerequisite in all ILLC separations. This is used to find the optimal flow rate for a given separation and to determine the optimal rotation speed that is required. This is carried out in the apparatus illustrated in Fig. 12, with recirculation of the fluids. The stationary phase retention curves are a plot of the fraction of stationary phase that remains in a coil with increasing flow rates of the mobile phase. The amount of stationary phase remaining in the coil is calculated by measuring changes in the position of the mobile phase–stationary phase boundary in a graduated measuring cylinder (Fig. 12). The level of the interface between the two phases in the solvent reservoir is used to calculate the percentage stationary phase retention (%* S*_f_) using Eq. **(** 1 **)** for the DPS mode and Eq. **(** 2 **)** for the LPS mode.

1 $$ \% \;S_{\text{f}} = \, 100 \, \times \, \left( {1 - \left[ {V{-}V_{0} {-}{\text{DV}}} \right] \, /V_{\text{c}} } \right) $$

for DPS

2 $$ \% \,S_{\text{f}} = \, 100 \, \times \, \left( {1 - \left[ {V_{0} {-}V + {\text{DV}}} \right] \, /V_{\text{c}} } \right) $$

for LPS, where *V* = the observed volume (in cm^3^) of the dense phase up to the interface between mobile and stationary phases in the measuring cylinder; *V*_0_ = observed volume (in cm^3^) of the dense phase up to the interface between the mobile and stationary phases in the measuring cylinder at the start of the experiment;*V*_c_ = volume of coil under test (in cm^3^) (see Table 3 for details). DV is the dead volume, which is the volume of the pipework, connectors, UV–Vis detector cell, connectors and valves (experimentally measured to be 4.0 cm^3^).

After setting the coil rotation rate (typically 300–865 rpm) and temperature (in the range 20–50 °C), the mobile phase is pumped in a stepwise manner (between 0.5 and 32 cm^3^ min^−1^), into the rotating coil at increasing flow rates completely flushed from the coil, or the measured coil inlet pressure exceeds 70 bar. The graph of flow rate versus % *S*_f_ is then plotted to give the stationary phase retention curve. This was carried out for the solvent systems in Tables 4 and 5.

**Table 5:** Solvent systems used in the generation of the stationary phase retention curves for the separations described in Table 4

Label	Stationary phase (mol%)	Mobile phase (mol%)	Solvent system	Ratio of solvent system components (v/v)
A	[C_12_mim][NTf_2_] (44%)/hexane (56%)	>99% hexane	[C_12_mim][NTf_2_]/hexane	1 3
B	[C_10_mim][OTf]/hexane	>99% hexane	[C_10_mim][OTf]/hexane	1 3
C	[C_4_mim]Cl (16%)/H_2_O (84%)	5.0 M K_2_[HPO_4_]_(aq)_	[C_4_mim]Cl/3.0 M K_2_[HPO_4_]	1 1
D	[P_6 6 6 14_]Cl/ethyl ethanoate	92.5% water, 7.5% ethyl ethanoate	[P_6 6 6 14_]Cl/ethyl ethanoate/water	1 1 4
E	5.0 M K_2_[HPO_4_] _(aq)_	[C_4_mim]Cl (16%)/H_2_O (84%)	[C_4_mim]Cl/3.0 M K_2_[HPO_4_]	1 1

**Fig. 14:**
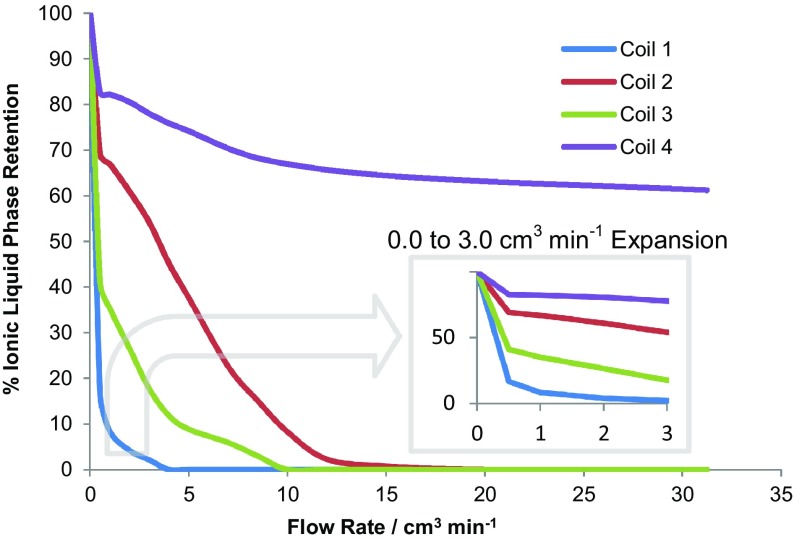
The stationary phase retention curves within the four coils (see Table 3) for solvent system D (see Tables 4, 5), at 35 °C and 865 rpm

**Fig. 15:**
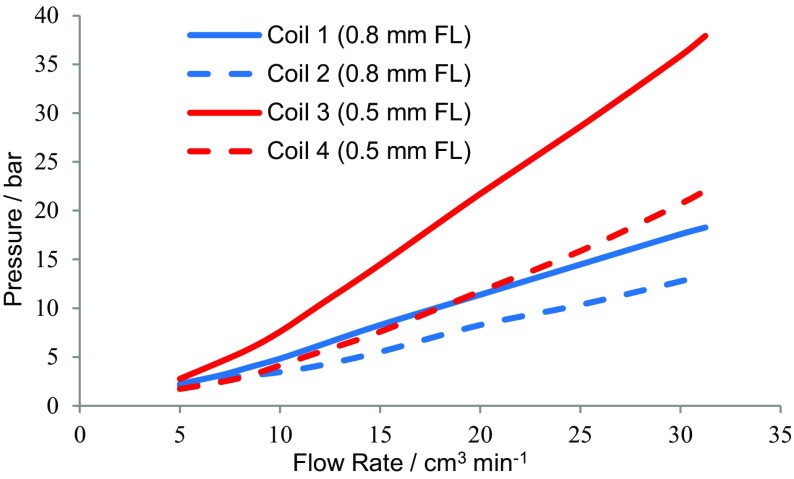
The mobile phase operating pressures encountered during the experiment to determine the phase retention curves in Fig. 14, for the water/[P_6 6 6 14_]Cl/ethyl ethanoate (4:1:1 v/v/v) solvent system with water as the mobile phase at 35 °C. Error = ±1.4 bar. *0.8 mm FL*,* 0.5 mm FL* Inner diameter of the polytetrafluoroethylene flying leads, respectively

For solvent system D (Tables 4, 5), the phase retention curves for all of the four coils (Table 5) were measured and calculated using Eq. **(** 2 **)**, at 865 rpm and 35 °C. The flow rate versus stationary phase retention curves were determined and are shown in Fig. 14. In addition, the coil inlet pressure was measured at each flow rate with an analogue pressure gauge (0–140 bar) (shown in Fig. 15). With the solvent system (D), it can be seen that much of the backpressure comes from the flying leads and that the 0.5-mm-bore tubing generates much higher backpressures. Measurements show that the pressure drop over the lengths of coil 2 and coil 4 is <1 bar at a 32 cm^3^ min^−1^ flow rate, so that the dashed lines in Fig. 15 show the pressure drop in the piping and flying leads of the IL-Prep™ CCC system.

In Fig. 14, it can be seen that there are considerable differences between the behaviour of the two liquid phases in the four coils (for solvent system D). The rate of loss of the ionic liquid stationary phase is dependent on both the coil diameter and length. For example, coils 1 and 3 have the same ID, but have completely different phase retention curves. This implies that the stationary phase is not evenly distributed in the coil during operation and is concentrated at the head of the coil. This observation would have to be accounted for in any computer modelling of the ionic liquid solvent systems used in this study. For good separations, the instrument should be operated at rotation speeds and flow rates that maximise the value of *S*_f_. Ideally, *S*_f_ values should be in the range of 60–95%. The best S_f_ values for D occur in the plateau region shown in the Fig. 14 inset. For coils 1 and 3, both of 1-mm bore (but different coil lengths), the best operating flow rates are at or below 0.5 cm^3^ min^−1^. However, these *S*_f_ values of 12% for coil 1 and 38% for coil 3 are too low to give a reasonable separation. For coil 2 of 2.1-mm bore, the best operating region is at 0.5–2.0 cm^3^ min^−1^ and for coil 4, 0.5–3.0 cm^3^ min^−1^. For the solvent system in coil 4, the separations can be run at very high flow rates, since the value of *S*_f_ drops to 60% at 32 cm^3^ min^−1^, which is a useable value.

**Fig. 16:**
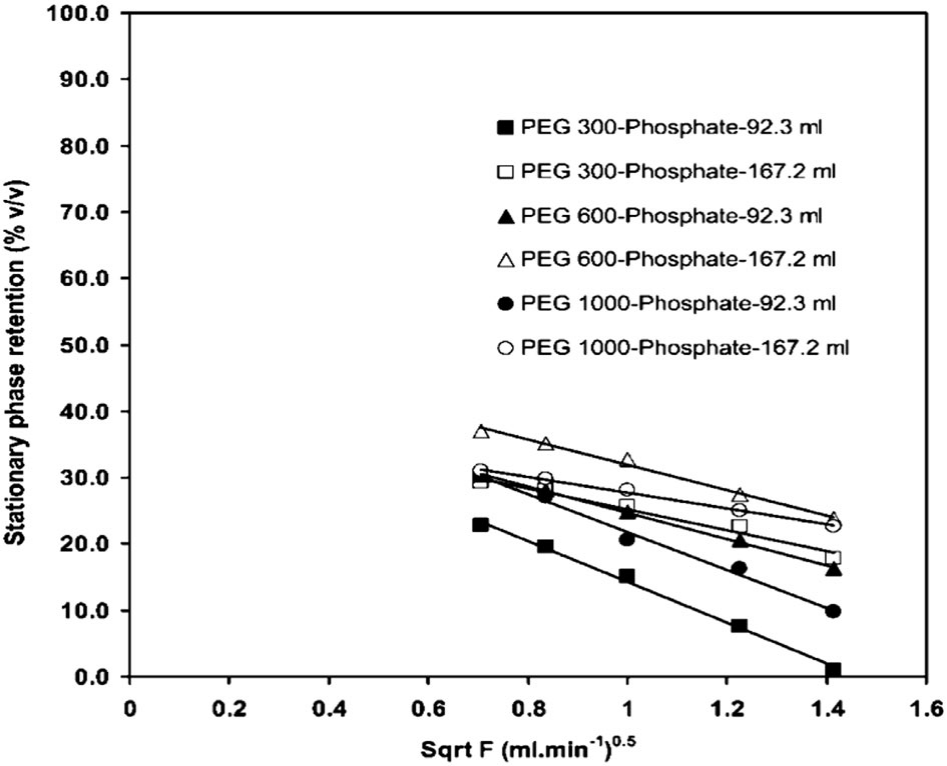
The stationary phase retention for various polyethylene glycol (*PEG*) molecular weights plotted against the square root of the mobile phase flow rate using column volumes of 92.3 cm^3^ (*closed symbols*) and 167.2 cm^3^ (*open symbols*). Rotational speed: 600 rpm; mobile phase: K_2_[HPO_4_] (18% w/w); stationary phase: PEG (18% w/w); direction: HEAD to TAIL. *Solid lines*: best fit linear regression [154]

**Fig. 17:**
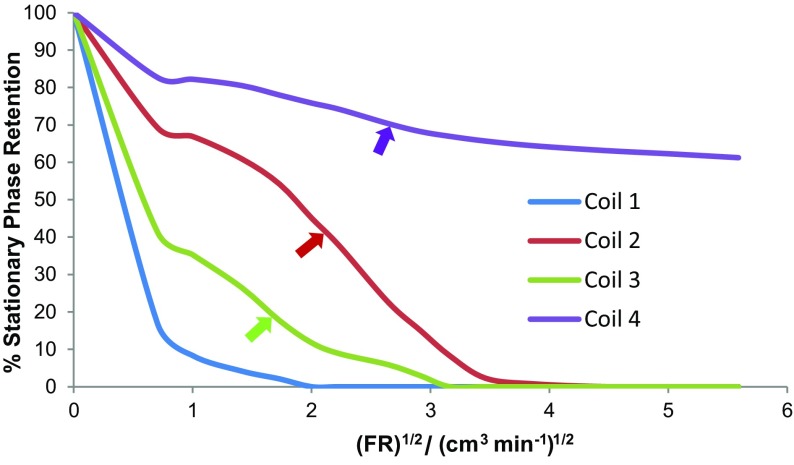
The stationary phase retention within the four coils (Table 3) plotted against the square root of flow rate (FR)^1/2^ for solvent system D, at 35 °C and 865 rpm. *Arrows* mark the points where the flow changes from laminar flow to emulsion flow of the two phases

For the water/ethanol/ethyl ethanoate/hexane solvent systems, a straight line in the graph of *S*_f_ versus $\sqrt {\text{FR}}$ occurs as the flow rate is increased, and therefore the relationship $S_{\text{f}} \propto \sqrt {\text{FR}}$ holds [146]. This relationship can also be seen in PEG–aqueous phosphate system shown in Fig. 16 [154]. The plot of the stationary phase retention versus the square root of flow rate for the water/[P_6 6 6 14_]Cl/ethyl ethanoate (4:1:1 v/v/v) solvent system (Table 4) is shown in Fig. 17. For this ionic liquid solvent system (Fig. 17), clearly the $S_{\text{f}} \propto \sqrt {\text{FR}}$ relationship does not describe the stationary phase retention curve.

Inflection point is defined as a point at which a change in the direction of curvature occurs. For all coils in Fig. 17, there is a common inflection point at 0.75 cm^3^ min^−1^. Additionally, there are inflection points at 3 cm^3^ min^−1^ (for coil 3), 5 cm^3^ min^−1^ (for coil 2) and 7 cm^3^ min^−1^ (for coil 4). Careful visual observation of the fluid exiting the coils at these flow rates reveals that there is a change from laminar flow to emulsion formation, which is most noticeable when the flow rate is increased [146].

The *S*_f_ of biphasic systems in the J-type centrifuge has been modelled by Wood et al. [25, 145, 155] and discussed by Berthod [156]. Generally high *S*_f_ values favour better separations. Unlike in conventional chromatography, in CCC any changes in the composition of one phase affects the composition and behaviour of the other phase [156]. In the metal(II) chloride separations that use water/[P_6 6 6 14_]Cl solvent systems, this can result in problems with the separation. For example, a solvent system in which the mobile phase is the least dense phase (normal phase CCC) will give good separations for small sample sizes (<0.5 g). However, for higher sample loadings of dense metal salts, the density of the mobile phase plus sample injected into the coil can exceed the density of the stationary phase (termed: phase inversion), resulting in the stationary phase being lost out of the end of the coil (termed: column bleed). To solve this problem, the separation is run in LPS mode with the mobile phase as the denser phase. The density of [P_6 6 6 14_]Cl is 0.882 g cm^−3^ [127], so any co-solvent used when combined with the ionic liquid must have a density of significantly less than the mobile phase density at the temperature of the separation (0.98 g cm^−3^ for water at 50 °C).

## Ionic Liquid–Liquid Separations

A range of separations using ionic liquid-containing solvent systems, where the ionic liquid is a major component of one of the phases, has been carried out. These separations illustrate the versatility of the methodology, allowing many compounds to be separated by a single technique, where the main variable is the structure and composition of the solvent system rather than the apparatus. Provided that a suitable two-phase solvent system can be designed and implemented, the number of possible separations that can be achieved with this technique is enormous. Here we show, as an illustration of the technique’s versatility, that ILLC can separate sugars, inorganic transition metal salts and low polarity organic molecules.

### Separation of Organic Compounds

For the separation of organic compounds, there are two types of solvent systems which could be used: (1) ionic liquid/non-polar solvent or (2) ionic liquid/polar solvent (commonly water). We present here examples of the separations of sugars, aromatics from simulated petroleum feedstock and natural oils.

#### Separation of Saccharides

The separation of simple sugars has been studied by various chromatographic techniques [157], but these are not widely used on an industrial scale [158]. There are however many HPLC methodologies that can be used for the separation of glucose and fructose from sucrose and other saccharides on an analytical scale [159], with refractive index detection [160] or UV–Vis detection [161]. Preparative HPLC separations of sugars are also possible [162], such as with the use of a PL-Hi-Plex Ca 300 × 25 mm preparative HPLC column with water as the eluent [163], but these suffer from the problem of small sample sizes (injection volume 30 μl) [163]. Sugar separations using CCC has been achieved using aqueous biphasic solvent systems, such as aqueous salt/ethanol systems, or the ethanenitrile/aqueous 1.0 M sodium chloride (5:4) system [164], but the systems were only tested on 2-mg sample sizes.

**Fig. 18:**
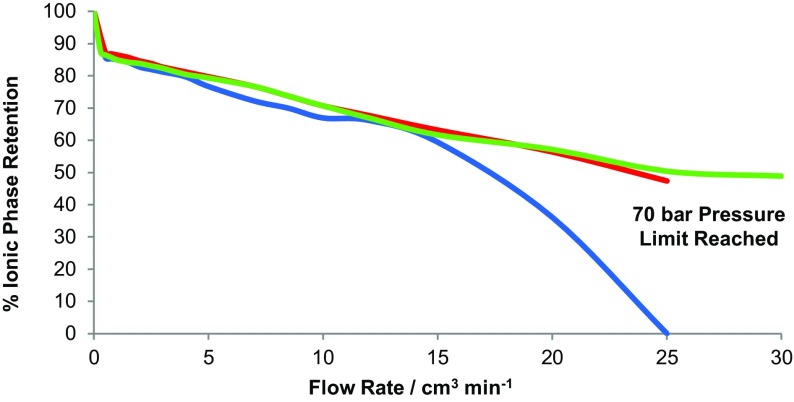
The variation of percentage stationary phase retention with respect to flow rate for the [C_4_mim]Cl/(K_2_[HPO_4_]–H_2_O; 3.0 M; 1:1) two-phase solvent system at 30 °C (*blue line*), 40 °C (*red line*) and 45 °C (*green line*), on coil 2 (see Table 3 for details)

The use of aqueous biphasic solvent systems based on chloride ionic liquids [109] has been tested in CCC by Berthod [117]. Here, it was possible to use the [C_4_mim]Cl/aqueous K_2_[HPO_4_] biphasic solvent systems in hydrostatic high-performance centrifugal partition chromatography (HPCPC) and hydrodynamic HPCCC instruments. However, with the J-type centrifuge with a volume of 53 cm^3^, containing a 26-m Teflon^®^ coil with an ID of 1.6 mm, operating at 500 or 900 rpm, only low stationary phase retention values were obtained [117]. These were typically <20%, and, in some cases, 0% at low flow rates [117]. The same biphasic system used in the IL-Prep machine (at 865 rpm and 30, 40 and 45 °C) gave very acceptable phase retention values of approximately 85% with a 2 cm^3^ min^−1^ flow rate (Fig. 18). This is thought to be due to the use of a longer (36 m) and wider bore (ID 2.1 mm) coil than the one described by Berthod [117] (see coil 2 in Table 3). As can be seen, the variation of temperature has little effect on the phase retention at a flow rate of <15 cm^3^ min^−1^. However, higher temperatures allow higher stationary phase retentions at faster flow rates, without exceeding the maximum operating pressure of the stainless steel coils and flying leads. The maximum rated operating pressure (70 bar) was exceeded at 30 and 40 °C, with a flow rate of 25 cm^3^ min^−1^. At 45 °C, the pressure limit is reached at 30 cm^3^ min^−1^ flow rate.

**Fig. 19:**
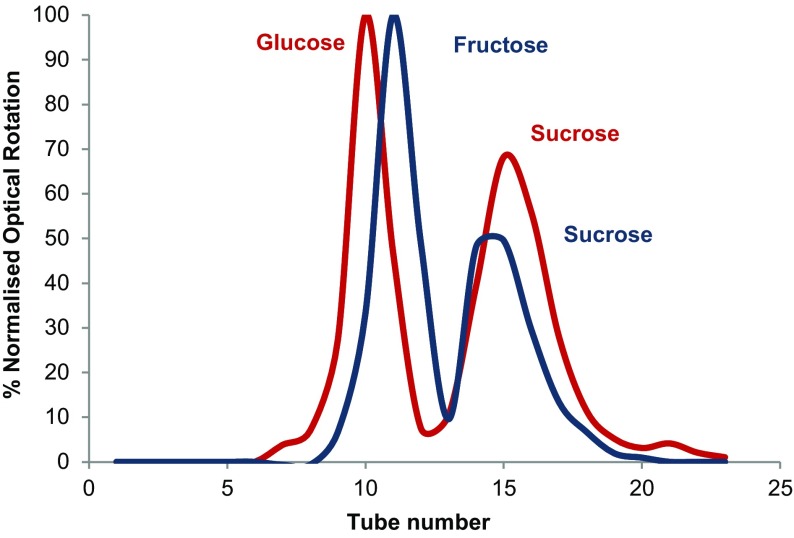
The separation of glucose (*red*) and fructose (*blue*) (fractions 7–12) from sucrose (fractions 13–20) by ILLC using the [C_4_mim]Cl/(K_2_[HPO_4_]–H_2_O; 3.0 M; 1:1 v/v) two-phase solvent system at 45 °C, on coil 2. Percentage normalised optical rotation = 100 × measured optical rotation/specific rotation for each sugar × normalising factor

The DR_*X*_ of sucrose, fructose and glucose in the [C_4_mim]Cl/(3.0 M aqueous K_2_[HPO_4_]; 1:1 v/v) two–phase solvent system was found to be 0.90, 0.22 and 0.26, respectively by ^1^H nuclear magnetic resonance (NMR) analysis. From the DR_*X*_ values, the separation of glucose from fructose would not be possible because they are too close to anticipate a reasonable separation; however, the separation of glucose or fructose from sucrose would be achievable. Since all three sugars are optically active, polarimetry can be used to quantitatively measure the concentration of sugars in fractions collected in the ILLC separation. The separation of glucose (0.5 g) from sucrose (0.5 g) (red line) and fructose (0.5 g) from sucrose (0.5 g) (dark blue line) is shown in Fig. 19.

It can be clearly seen that complete separation of the two monosaccharides from the disaccharide (sucrose) was achieved working at a preparative scale (1.0 g). This separation is a proof-of-principle demonstration of the large increase of scale (from 2 to 1000 mg) that can be achieved with an ILLC system over previous CCC separations of sugars with aqueous biphasic solvent systems [164]. In practice, the separation of fructose or glucose from sucrose is achieved on an industrial scale using crystallisation techniques, and chromatographic techniques are not economical [165]. From a more application-orientated viewpoint, this same ILLC solvent system has been used successfully on high value saccharides, oligosaccharides and polysaccharides, the details of which are available elsewhere [42].

#### Aromatic/Alkane Separations

**Fig. 20:**
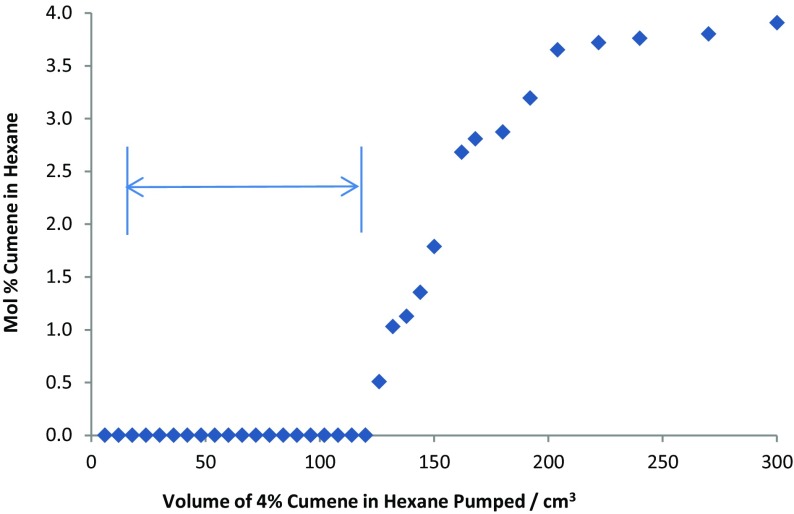
Extraction of cumene from hexane on coil 4 using the ionic liquid [C_10_mim][OTf]. The part of the graph from 0 to 11 cm^3^ corresponds to hexane (initially contained within the coil) being pumped out of the coil, and the region of 11–110 cm^3^ shows hexane which has been completely stripped of cumene

The extraction of aromatics from petroleum was modelled by using a simulant comprising of a solution of cumene (isopropylbenzene; 4 mol%) in hexane. A two-phase system was produced consisting of [C_10_mim][OTf], mixed with hexane, with the ionic liquid phase used as the stationary phase and the hexane used as the mobile phase. Coil 4 was filled with the stationary phase, followed by the mobile phase (hexane) at 2 cm^3^ min^−1^, with the coils rotating at 865 rpm, resulting in stationary phase retention of 95%. The analyte was pumped into the coil, and aliquots (5 cm^3^) were collected in the fraction collector. Offline analysis by ^1^H NMR spectroscopy of the concentration of cumene in the collected fractions is shown in Fig. 20. As can be seen, the aromatics were completely captured by the stationary ionic phase, until the ionic liquid in the coil became saturated, and breakthrough of the cumene occurred at 110 cm^3^. The stationary phase containing cumene was then back-flushed from the coil with fresh stationary phase, resulting in a transfer of cumene from hexane to the ionic phase. The cumene was then recovered by distillation from the ionic liquid, effecting a separation of cumene from hexane. This process has the potential to be automated using simulated moving bed (SMB) technology [166, 167] and provides a means for the complete separation of aromatics from alkanes.

#### Separation of Vetiver Oil

**Fig. 21:**
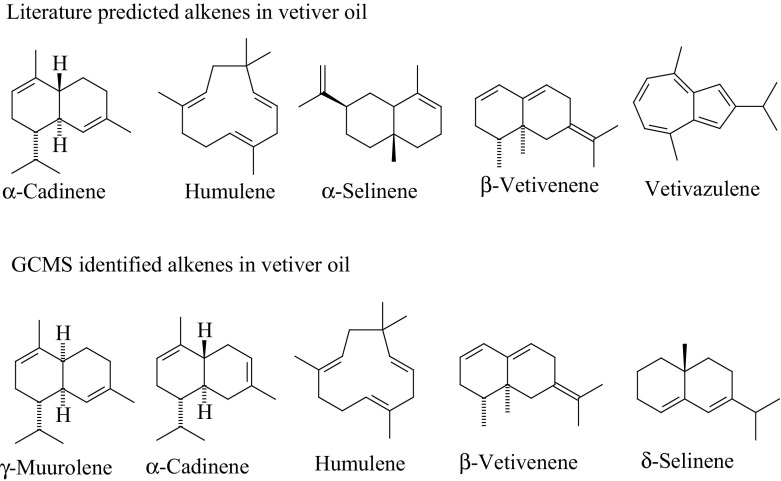
Five literature examples of alkenes present in vetiver oil and five compounds found in tubes 6–29 in the ILLC separation of vetiver oil, identified by GC-MS software [173]

Vetiver oil is extracted from the roots of *Chrysopogon zizanioides* [168, 169] by steam distillation [170], with a worldwide production of about two hundred and fifty tons per year [171]. The oil is widely used in the fragrance industry and is comprised of over three hundred compounds [172]. Constituents of interest to the fragrance industry include polycyclic alkenes (such as α-cadinene and β-vetivenene, as in Fig. 21) and polycyclic alcohols, such as khusimol [173]. Although separation into each individual component is not practical, or even desired, a rough separation into two main classes of 15 carbon sesquiterpenes can be achieved using only one run of ILLC.

**Fig. 22:**
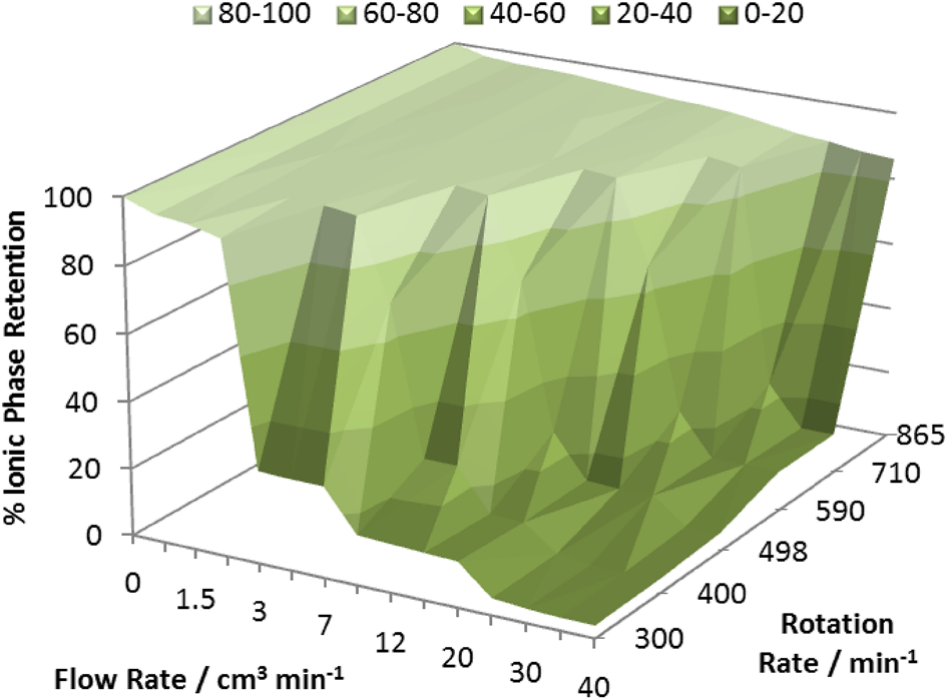
The variation of % stationary phase retention with respect to coil rotation rate and flow rate for the hexane (mobile phase)/[C_12_mim][NTf_2_] (stationary phase) (3:1) biphasic solvent system at 30 °C on coil 4 (Table 5)

The separation was carried out using a solvent system based on the [C_12_mim][NTf_2_]/hexane [124]. The ionic liquid is insoluble in the hexane phase (less than 0.1 mol% and could not be detected by ^1^H NMR spectroscopy), however hexane is soluble in the [C_12_mim][NTf_2_] phase (56 mol%, or 21 vol% or 13 wt%, as determined by ^1^H NMR spectroscopy). The stationary phase retention curves of the [C_12_mim][NTf_2_]/hexane solvent system (Table 4), using the ionic liquid phase as the stationary phase and hexane as the mobile phase (using the DPS operating mode as described in Table 4), were determined at a range of different rotation speeds, and are shown in Fig. 22. For the best separations, the instrument should be operated with high stationary phase retentions in the lightest green region or plateau region (where *S*_f_ = 85–95%) on the graph. The lower rotation speeds reduce wear on the instrument and reduce power consumption, while in this case giving rise to good phase retention values at speeds as low as 300 rpm and flow rates at or below a 2 cm^3^ min^−1^. These are very high stationary phase retention values, and hence there exists the potential to carry out very fast separations at high flow rates.

It is noticeable that the stationary phase retention curves of this solvent system are more complex than those encountered by He and Zhao [146]. The relationship $\%  S_{f} = 100 + k  \sqrt {\text{FR}}$ (where *k* = −2.20 $\pm$ 0.13, *R* ^2^ = 0.98) holds only for the plateau region of the graph, and it was found that the *S*_f_ value was independent of the rotation rate in rpm.

**Fig. 23:**
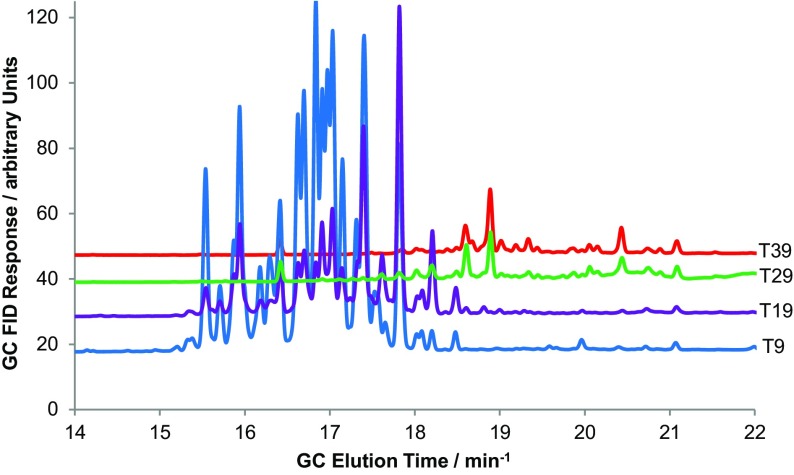
The gas chromatography (GC) response for selected fractions (*T9*,* T19*,* T29* and* T39*, where* T* is tube number) in the separation of vetiver oil with a hexane/[C_12_mim][NTf_2_] biphasic solvent system using hexane as the mobile phase. Tube numbers 6 to 25 contain C_15_ hydrocarbon terpenes (alkenes) and tube numbers >26 contain predominately oxygenated C_15_ terpenes.* FID* Flame ionisation detector

The vetiver oil (1.0 g) dissolved in mobile phase (4 cm^3^) was injected at 2 cm^3^ min^−1^ into Coil 2 (*S*_f_ = 95%) and 106 fractions (5.0 cm^3^) were collected. A representative set of fractions were collected and analysed by GC–MS, and four of the GC traces are shown in Fig. 23. The GC analysis of the fractions revealed that the vetiver oil sample was composed of sesquiterpenes, which were separated into two main classes [174, 175]. The fractions in the T6-T25 range contained sesquiterpene alkenes with the formula C_15_H_24_ or C_15_H_26_ [175]. Examples include α-muurolene, β-cadinene, humulene, β-vetivenene and δ-selinene (Fig. 21). From fraction T26 onwards, the compounds isolated were mostly oxygenated hydrocarbons such as ethers, epoxides and alcohols, eluting in order of increasing distribution ratio. The chemical formula of these oxygenated products were C_15_H_24_O and C_15_H_26_O by GC-MS analysis. This isocratic ILLC process can therefore be used to carry out a sesquiterpene alkene from oxygenated sesquiterpene separation.

### Separation of Transition Metal Salts

#### Previous Research

Aqueous copper(II), nickel(II) and cobalt(II) chloride can be separated from each other using a diverse range of techniques. On the preparative scale, examples include a complex nine-stage process [176] involving a combination of solvent extraction and electro-refining. Nickel(II) and cobalt(II) chloride have been separated by a five-stage continuous process using the ionic liquid [P_6 6 6 14_]Cl [177]. The chromatographic separation of these metal(II) salts has been carried out using a number of techniques, including anion-exchange chromatography [178], adsorption thin layer chromatography (TLC) of metal(II) chelates [179], TLC [180], HPLC of diaminostilbene complexes [181], chromatographic separation of amino acid chelates [182], micellar electrokinetic chromatography [183], a guar-based chelating ion exchange resin chromatography [184], and a dibenzyl sulfoxide solution supported on silica as a stationary phase [185].

The direct single-step preparative chromatographic separation of these metal(II) salts, with high sample loadings, without losing any metal(II) salts and without using metal(II) chelates or corrosive acids, would represent a significant improvement on these many processes. Hence, based on knowledge of the speciation of Ni(II), Co(II), and Cu(II) chloride ionic liquids and with the use of a hydrophobic chloride ionic liquid, a biphasic solvent system based on water and [P_6 6 6 14_]Cl (a hydrophobic ionic liquid) was tested and found to be suitable for ILLC separation.

#### Transition Metal Behaviour in the Water/[P_6 6 6 14_]Cl/Ethyl Ethanoate Solvent System

**Fig. 24:**
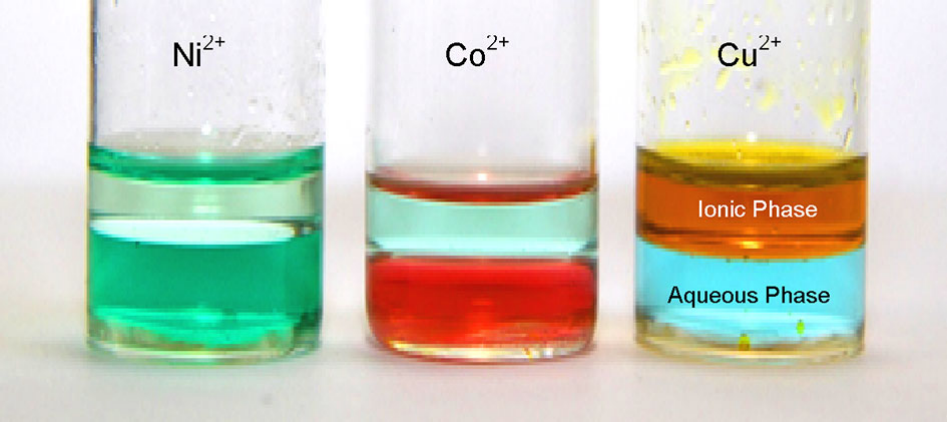
Solutions of nickel(II) (*Ni* ^*2+*^) chloride (NiCl_2_, *left*), cobalt(II) (*Co* ^*2+*^) chloride (CoCl_2_ *, centre*) and copper(II) (*Cu* ^*2+*^) chloride (CuCl_2_, *right*) dissolved in a biphasic mixture of water/[P_6 6 6 14_]Cl/ethyl ethanoate in the ratio of 4:1:1 v/v/v. The upper phase is the ionic liquid [P_6 6 6 14_]Cl and ethyl ethanoate and the lower phase is water, saturated with ethyl ethanoate. In the aqueous phase, the metals exist as octahedral [M(H_2_O)_6_]^2+^ [186] (Fig. 25), and in the chloride ionic liquid phase, the tetrahedral [MCl_4_]^2−^ complex is formed (Fig. 25)

**Fig. 25:**
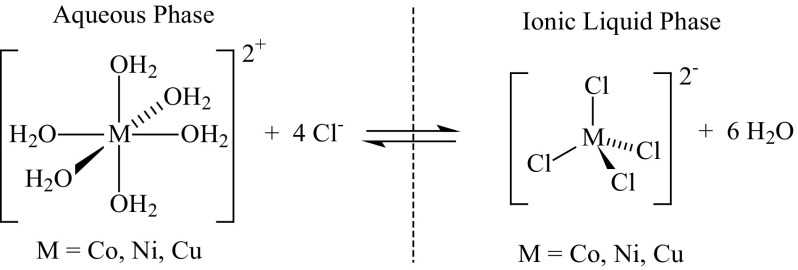
The equilibria between metal(II) chloride complexes (Co, Ni, or Cu) in the aqueous phase and in the chloride ionic liquid phase

**Fig. 26:**
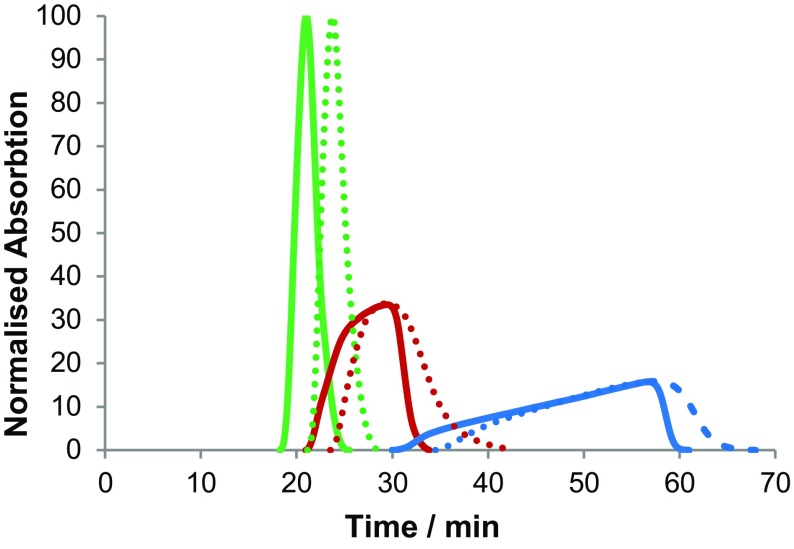
The concentration of aqueous cobalt(II) (*red line*), nickel(II) (*green line*) and copper(II) (*blue line*) at 30 °C (*dotted lines*) and 40 °C (*solid lines*) on coil 2, showing the normalised absorption of the metal halide salts where aqueous NiCl_2_ = 100 (equal areas under each curve). Error = ± 2% in UV absorption readings

When nickel(II), cobalt(II) or copper(II) chloride are dissolved in water, they form the well known hydrated [M(H_2_O)_6_]Cl_2_ salts (where M = Co, Ni or Cu) [186]. If this solution is mixed with a water-immiscible chloride salt such as the ionic liquid [P_6 6 6 14_]Cl, a biphasic solution is formed (shown in Fig. 24) [40]. In the chloride ionic liquid phase, the metals are presumed to exist as the tetrahedral [MCl_4_]^2−^ complexes (see Fig. 25) [187–189]. The three metals distribute themselves between both phases, with the nickel(II) and cobalt(II) salts both preferring to dissolve mostly in the aqueous phase, and the copper(II) salt preferring the ionic liquid phase. The distribution ratios of the metals between the water and the [P_6 6 6 14_]Cl/ethyl ethanoate phases can be estimated from the graph of the metal(II) chloride separation in Fig. 26, or can be derived by UV–Vis spectrophotometry of the metal(II) chlorides dissolved in each of the two phases. These DR_*X*_ are shown in Table 6. The values calculated from the peak maximum heights differ slightly from the values obtained from single metal(II) chloride salts obtained by UV–Vis spectroscopy.

**Table 6:** The distribution ratios calculated from the elution time of the metal(II) complexes described in Section 5.2.3 and from UV–Vis spectrophotometry

Metal complexes	Temperature (°C)	Peak maximum/min^a^	Distribution ratio from ILLC separation	Distribution ratio from UV–Vis spectrophotometry
Ni	30	23.5	0.31	0.2
Co	30	29.5	0.62	0.6
Cu	30	56.8	2.22	2.2
Ni	40	20.8	0.16	0.2
Co	40	29.8	0.66	0.6
Cu	40	55.3	2.07	2.1

^a^Solvent front elution time occurred at 18 min

The mobile aqueous phase is composed of water saturated with ethyl ethanoate (7.6 wt% at 30 °C and 7.2 wt% at 40 °C) [190]. The recovery of the metal halide salts from this phase involves simply evaporation of the mobile phase. The ionic liquid used in the stationary phase is [P_6 6 6 14_]Cl, which is a viscous, hydrophobic ionic liquid. The viscosity of 98% pure [P_6 6 6 14_]Cl (with 115 ppm water content) is 2161 cP at 298.15 K [191], which is too high to be pumped using standard HPLC pumps. Hence, the [P_6 6 6 14_]Cl ionic liquid had to be diluted with a water immiscible co-solvent, such as ethyl ethanoate. Other co-solvents tested include dichloromethane, hexane, propanone, butan-1-ol, butanone and pentan-2-one, all of which were also found to be effective co-solvents, with pentan-2-one giving the best separations. Typically, the scale of the separations was 0.5–6.0 g (at approx. 0.6–7.0 M concentration dissolved in the mobile phase) on coil 2 and 1–10 g on coil 4 (see Table 3 for coil details).

#### Transition Metal Separation

To achieve an effective separation of the aqueous metal(II) chloride salts [M(H_2_O)_6_]Cl_2_ (where M = Co, Ni or Cu), a biphasic solvent system (degassed) consisting of [P_6 6 6 14_]Cl (250 cm^3^), ethyl ethanoate (250 cm^3^) and deionised water (1000 cm^3^) was prepared. The ionic phase was found to contain the majority of the ethyl ethanoate, and forms the upper liquid layer. The aqueous phase of water (92.4–92.8 wt%) and ethyl ethanoate (7.2–7.6 wt%) was analysed by ^1^H NMR and found not to contain the ionic liquid [P_6 6 6 14_]Cl (the concentration of [P_6 6 6 14_]Cl in the mobile water phase was not detectable and estimated to be < 0.5 mol% concentration by this technique) [144].

**Fig. 27:**
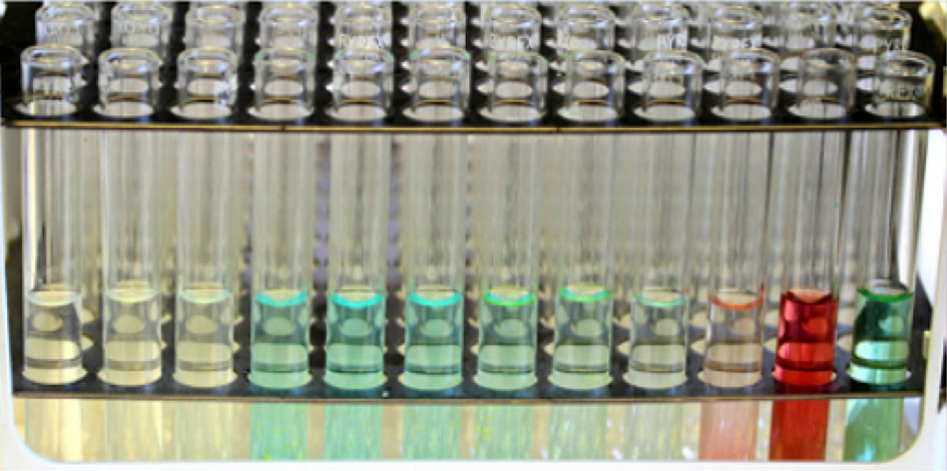
The separation of aqueous CoCl_2_ (*pink*/*red*), NiCl_2_ (*dark green*) and CuCl_2_ (*light blue*) by ILLC as viewed in the fraction collector, showing tubes 13 (*right*) through to 24 (*left*) in sequence

The separation of the copper(II), nickel(II) and cobalt(II) aqueous complexes dissolved in water was analysed by means of a UV–Vis detector situated before the fraction collector. A mixture of metal halides (approx. 2.9 M of MCl_2_ in the aqueous mobile phase, where M = Ni, Co or Cu) was prepared from NiCl_2_, CoCl_2_ and CuCl_2_ (a mixture of 0.50 g of each metal salt, dissolved in 5.0 cm^3^ of mobile phase). The separation was performed on coil 2 at 30 and 40 °C, with a mobile phase flow rate of 2.0 cm^3^ min^−1^. The raw data obtained from the UV–Vis detector were somewhat noisy and contained a number of spurious peaks due to tiny bubbles of stationary phase passing through the detector. The data were processed by computer subtraction of the baseline signal (at 590 nm, where the three metal salts do not absorb light) from the Ni(II) (390 nm), Co(II) (505 nm) and Cu(II) (790 nm) signals. This had the effect of eliminating baseline drift, removing the artifacts due to stationary phase bubbles passing through the detector cell and increasing the signal-to-noise ratio. Next, the data were smoothed with a moving average filter (taking the average over 42 s of data), the start of each metal peak was set to zero and a linear baseline adjustment was made to each peak to adjust the end of the peak to zero. The peak areas were normalised and equalised for each metal, compensating for the different molar absorption coefficients, to give the curves shown in Fig. 26. The separation of these three metal salts is visually demonstrated in Fig. 27, where the three separated metal salts are clearly distinguishable in the fraction collector.

At 30 °C, a partial separation was achieved with some of the cobalt(II) chloride ending up mixed with the copper(II) and nickel(II) chloride eluents. At 40 °C the separation performance improved (see Table 7; Fig. 26), and the separation time was reduced from 68 to 61 min. In Table 7, the area of overlap of the peaks and the percentage of pure unmixed metal chloride salt that can be obtained are shown. These figures have been obtained on a non-optimised system and are illustrative of the potential of this technique. The peak shape of the copper(II) complex in Fig. 26 is unusual and is showing anti-Langmuirian behaviour [192]. This is largely due to the presence of nickel(II) and cobalt(II) complexes affecting the phase equilibria of the copper(II) complexes, leading to the observed triangular peak shape.

**Table 7:** Percentage area of overlap of nickel(II)/cobalt(II) peaks and cobalt(II)/copper(II) peaks at 30 and 40 °C and the percentage integration of pure nickel(II), cobalt(II), and copper(II) chlorides eluted (area of each peak that does not overlap)

Temperature (°C)	% Area overlap nickel(II)/cobalt(II)	% Area overlap cobalt(II)/copper(II)	% Pure nickel(II)	% Pure cobalt(II)	% Pure copper(II)
30	14.7	3.98	36	57	88
40	10.7	1.26	48	73	98

## Conclusions

The use of ionic liquids in ILLC was originally thought to be too difficult to be of use in conventional CCC equipment. With the focussed design of the equipment by AECS-QuikPrep Ltd., this problem has now been solved. Currently there is very little literature precedent for the use of ionic liquids in CCC or LLC, and so much of this technology has had to be developed from first principles. This has required the designing of ionic liquids and solvent systems which give acceptable distribution ratios for the solutes being separated. The testing of these new solvent systems has been carried out in a specially designed ILLC instrument which can handle higher viscosity solvent systems than are usually found in CCC and LLC. Despite this, ILLC has been shown to be a very versatile technology which allows a diverse range of separations, including the direct separation of both organic and inorganic systems. The instrumentation for ILLC separations is closely related to conventional HPCCC/HSCCC equipment, but uses coils that are made from stainless steel (rather than PTFE), and the bottlenecks in the solvent flow pathways have been removed.

Ionic liquids can be designed to suit a particular separation by modifying their structure and therefore their properties. Factors such as viscosity, density, density difference, interfacial tension and relative solubility of solutes can all be adjusted. Thus, this technique allows the separation of practically any soluble mixture, provided a suitable two-phase solvent system can be designed or found. One way of regarding ILLC is as a method of using “designer solvents for designer separations”. Whilst still in its infancy, ILLC research should have significant impact on separations which are currently either too difficult or too expensive to perform at scale. Compounds thought to be too insoluble or too immiscible with biphasic solvent systems comprised of molecular solvents can now be separated by ILLC. One such example is the separation of lentinan [42], where the scale of separation was boosted by a factor of 100, from the tens of milligrams to the gram scale, on a similar sized apparatus, by replacement of a water/PEG biphasic solvent system with an ionic liquid/aqueous phosphate solvent system. With larger capacity ILLC instruments and through the use of CPC instrument designs, much larger scale separations could be achieved. In Table 8, the versatility and relative advantages of ILLC (in comparison with conventional HPCCC, HPLC and GC) methodologies are shown, and it is clear that ILLC offers major benefits.

**Table 8:**
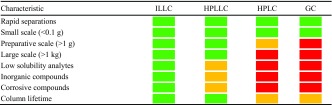
The comparison of ionic liquid–liquid chromatography, high-performance liquid–liquid chromatography, high-performance liquid chromatography and gas chromatography systems

Colour codes: *red* poor, *yellow* intermediate, and *green* good

## Abbreviations

ABS: Aqueous biphasic solvents
CCC: Countercurrent chromatography
CPC: Centrifugal partition chromatography
[C_*n*_mim]: 1-Alkyl-3-methylimidazolium, where *n* = alkyl chain length
[C_*n*_py]: 1-Alkylpyridinium, where *n* = alkyl chain length
[N_111_(C_2_OH)]^+^: Cholinium or 2-(hydroxyethyl)trimethylammonium
DR_*X*_: Distribution ratio (of component *X*)
DMSO: Dimethyl sulfoxide
DPS: Dense phase stationary (in biphasic solvent systems)
GC: Gas chromatography
HEMWat: Hexane, Ethyl ethanoate, Methanol, Water
HPCCC: High-performance countercurrent chromatography
HPLC: High performance liquid chromatography
HPLLC: High performance liquid–liquid chromatography
HSCCC: High-speed countercurrent chromatography
IL: Ionic liquid
ILLC: Ionic liquid–liquid chromatography
LLC: Liquid–liquid chromatography
LPS: Light phase stationary (in biphasic solvent systems)
MP: Mobile phase
NTf_2_: Bis{(trifluoromethyl)sulfonyl}amide or [N(SO_2_CF_3_)_2_]^−^
OTf: Trifluoromethanesulfonate or [SO_3_CF_3_]^−^
PEG: Polyethylene glycol
SP: Stationary phase
*S*_f_: Stationary phase retention factor
[P_6 6 6 14_]^+^: Trihexyltetradecylphosphonium cation
VOCs: Volatile organic compounds
